# Dynamic RNA Regulation in the Brain Underlies Physiological Plasticity in a Hibernating Mammal

**DOI:** 10.3389/fphys.2020.624677

**Published:** 2021-01-18

**Authors:** Rui Fu, Austin E. Gillen, Katharine R. Grabek, Kent A. Riemondy, L. Elaine Epperson, Carlos D. Bustamante, Jay R. Hesselberth, Sandra L. Martin

**Affiliations:** ^1^RNA Bioscience Initiative, University of Colorado School of Medicine, Aurora, CO, United States; ^2^Fauna Bio Incorporated, Emeryville, CA, United States; ^3^Department of Biomedical Data Science, Stanford University, Stanford, CA, United States; ^4^Center for Genes, Environment & Health, National Jewish Health, Denver, CO, United States; ^5^Department of Biochemistry and Molecular Genetics, School of Medicine, University of Colorado, Aurora, CO, United States; ^6^Department of Cell & Developmental Biology, School of Medicine, University of Colorado, Aurora, CO, United States

**Keywords:** AU-rich element (ARE), ARE binding proteins, forebrain, hypothalamus, *Ictidomys tridecemlineatus*, medulla, pre-miRNA, RNA binding protein

## Abstract

Hibernation is a physiological and behavioral phenotype that minimizes energy expenditure. Hibernators cycle between profound depression and rapid hyperactivation of multiple physiological processes, challenging our concept of mammalian homeostasis. How the hibernator orchestrates and survives these extremes while maintaining cell to organismal viability is unknown. Here, we enhance the genome integrity and annotation of a model hibernator, the 13-lined ground squirrel. Our new assembly brings this genome to near chromosome-level contiguity and adds thousands of previously unannotated genes. These new genomic resources were used to identify 6,505 hibernation-related, differentially-expressed and processed transcripts using RNA-seq data from three brain regions in animals whose physiological status was precisely defined using body temperature telemetry. A software tool, squirrelBox, was developed to foster further data analyses and visualization. SquirrelBox includes a comprehensive toolset for rapid visualization of gene level and cluster group dynamics, sequence scanning of *k*-mer and domains, and interactive exploration of gene lists. Using these new tools and data, we deconvolute seasonal from temperature-dependent effects on the brain transcriptome during hibernation for the first time, highlighting the importance of carefully timed samples for studies of differential gene expression in hibernation. The identified genes include a regulatory network of RNA binding proteins that are dynamic in hibernation along with the composition of the RNA pool. In addition to passive effects of temperature, we provide evidence for regulated transcription and RNA turnover during hibernation. Significant alternative splicing, largely temperature dependent, also occurs during hibernation. These findings form a crucial first step and provide a roadmap for future work toward defining novel mechanisms of tissue protection and metabolic depression that may 1 day be applied toward improving human health.

## Introduction

**FIGURE 1:**
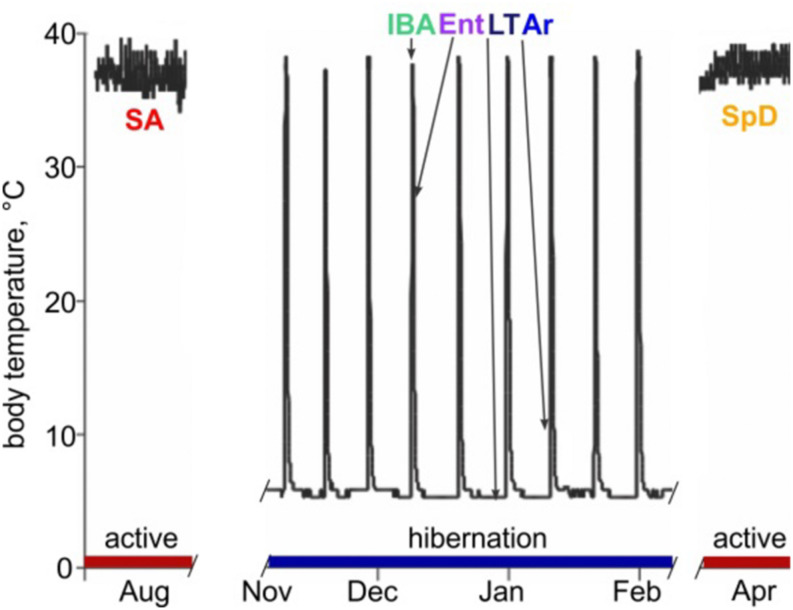
Abdominal body temperature and hibernation behavior in 13-lined ground squirrels. Schematic of Tb vs. time shows heterothermic (hibernation, blue) and homeothermic (active, red) portions of a year and physiological states sampled for RNA-seq: SA, summer active; IBA, interbout aroused; Ent, entrance; LT, late in a torpor bout; Ar, arousing from torpor; SpD, spring dark. See Section “Materials and Methods” for additional details.

Hibernating mammals exhibit extreme and highly predictable physiological plasticity across daily, seasonal and annual cycles. Most impressively, they temporarily suspend the mammalian trait of homeothermy by suppressing metabolic heat production. This block allows body temperature (Tb) to drop to near freezing, and the animal to enter a state of deep torpor. By spending much of their fall and winter months in torpor, hibernators achieve profound energetic savings compared to what would be required to maintain Tb at 37°C when environmental temperatures are near or below freezing (reviewed in Staples, 2016). It is noteworthy that torpor is typically discontinuous over the period of hibernation; prolonged bouts (days to weeks) of torpor are periodically and regularly interrupted by short periods (<24 h) of energy-intense reactivation of metabolism and Tb called interbout arousal (IBA). Cycles between torpor and IBA give rise to a pattern of seasonal heterothermy (Figure 1). Although hibernation patterns among mammals vary in detail (van Breukelen and Martin, 2015), the behavior typically occurs on a near annual, i.e., circannual, cycle. *Ictidomys tridecemlineatus* (13-lined ground squirrels) alternate between a spring and summer active period of reproduction, growth and fattening, and the fall and winter hibernation period (Carey et al., 2003).

The extraordinary phenotypic dynamics of the mammalian hibernator is orchestrated via a pattern of differential gene expression (Srere et al., 1992). In circannual hibernators, like the 13-lined ground squirrel, at least two key phenotypic cycles comprise hibernation (Epperson et al., 2011). The first is the seasonal cycle between permissive and not permissive for torpor, which enhances fat utilization (Martin, 2008), neuron function in the cold (Hoffstaetter et al., 2018) and tissue protection, including neuroprotection (Frerichs and Hallenbeck, 1998; Schwartz et al., 2015), during the many months of hibernation. The second is the cycle between the state of profoundly suppressed metabolic activity that results in cold Tb during torpor, and the rapid, intense physiologic and metabolic activation that rewarms the animal in IBA (Figure 1). Importantly, the animals first slow metabolic rate to reversibly enter the torpid state. Likewise, they must first reactivate metabolism despite very low Tb to rewarm for each interbout arousal (Wilz and Heldmaier, 2000). Mechanisms that protect cells, tissues and organs from damage due to the profound fluctuations of Tb and oxygen delivery, especially during the hyperactive metabolism that drives Tb recovery at the end of a torpor bout (i.e., during arousal from torpor), are likely equally critical for the hibernating phenotype. We expect that the molecular mechanisms underlying metabolic suppression and reactivation, as well as those responsible for seasonal tissue protection, will be revealed by identifying differentially-expressed (DE) genes among the distinct physiological stages of these two phenotypic cycles.

While a full understanding of hibernation will require defining differential expression in tissues throughout the body, here we focus on three brain regions: forebrain, hypothalamus and medulla. The forebrain is quiescent during entrance into torpor and remains so throughout the torpor bout and as animals initiate arousal from torpor (Kilduff et al., 1990). Despite inactivity, forebrain neurons undergo morphological changes during the prolonged period at low Tb that are rapidly reversed on rewarming (von der Ohe et al., 2006). In contrast, neuronal activity is more apparent in the hypothalamus and medulla as animals enter, maintain and arouse from torpor (Kilduff et al., 1990). This activity is consistent with the importance of at least a subset of neurons in both of these areas for autonomic functions with key roles in the torpor-arousal cycle, including body temperature, metabolic, respiratory and heart rate control (Bratincsák et al., 2007; Russell et al., 2019).

Few studies to date have attempted to discover the molecular mechanisms of hibernation or torpor in the brain using RNA-seq. The earliest considered two brain regions, cortex and hypothalamus, and four collection points from 13-lined ground squirrels: two from active animals before and after hibernation (October and April) plus torpid and interbout-aroused hibernators (Schwartz et al., 2013). But the majority of studies consider simply paired comparisons of winter torpid hibernators vs. summer active. These paired RNA-seq studies have been done using whole brain from *Rhinolophus ferrumequinum* (greater horseshoe bats) (Lei et al., 2014) and *Dromiciops gliroides* (Monito del Montes, a marsupial Nespolo et al., 2018), as well as medullary respiratory centers in pre-adapted vs. winter adapted *Mesocricetus auratus* (Syrian hamsters, Russell et al., 2019). Hypothalamus gene expression between animals entering daily torpor vs. those remaining euthermic in winter-adapted *Phodopus sungorus* (Djungarian hamsters) have also been compared by RNA-seq (Cubuk et al., 2017). All of the above studies were limited by infrequent and imprecise sampling across the phenotypic complexity of hibernation, small sample sizes, and incomplete genomes with sparse annotation.

To address these limitations, we first improved the 13-lined ground squirrel genome and its annotation and then used this enhanced genomic resource to define quantitative and qualitative changes in the transcriptome. Key to this study are the use of samples for RNA-seq that were precisely collected from ground squirrels in four physiologically distinct phases of hibernation based on body temperature telemetry, in addition to the two sample groups from the active phase that bracket hibernation. Our sample collection strategy (Epperson et al., 2011), combined with a relatively large sample size (*n* = 5 individuals in each group) and enhanced 13-lined ground squirrel genome and annotation, allow separation of seasonal and torpor-arousal effects on gene expression for the first time. These data highlight the importance of precise sample collection for discovery research on hibernation and provide strong evidence for both season and temperature-related effects on the transcriptome. As initially discovered in a study of brown adipose tissue (Grabek et al., 2015), a subset of transcripts in all three brain regions are particularly stabilized across the torpor bout when transcription effectively ceases (van Breukelen and Martin, 2002). We propose a model whereby the surviving transcripts set up a transcriptional cycle that instructs torpor-arousal cycle dynamics.

## Results

### Improved 13-Lined Ground Squirrel Genome and Annotation

**FIGURE 2:**
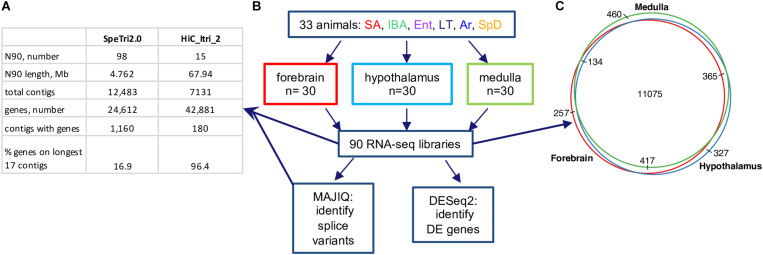
13-lined ground squirrel genome and RNA-seq data. **(A)** Comparison of original (SpeTri2.0) and new genomes. Note that there are 16 autosomes in the 13-lined ground squirrel (Lemskaya et al., 2018) and the sequenced individual was female. **(B)** Schematic of RNA-seq data, collection and use. For each brain region the 30 animals comprised *n* = 5 individuals from each of the six physiological groups defined in Figure 1; DE, differential expression. **(C)** Transcripts for most genes (11075/13035, 85%) were detected in all three brain regions.

Sensitive differential expression testing requires an accurate and complete transcriptome annotation. For this study on gene expression dynamics in hibernation, we built an improved 13-lined ground squirrel genome and annotated it using a novel pipeline (see section “Materials and Methods”). The new genome, HiC_Itri_2, exhibits little change in its total length of 2.5 Gb compared to the publicly available SpeTri2.0. The contiguity of the new genome is greatly improved, however, having just 15 N90 scaffolds of 67.94 Mb relative to the SpeTri2.0 N90 with 98 scaffolds of 4.762 Mb. The NCBI annotation (release 102 gff file) reports 24,612 genes across 1,160 SpeTri2.0 scaffolds for the 13-lined ground squirrel. We combined the 21,998 best-annotated genes from the NCBI transcriptome with 6,527 non-overlapping Ensembl annotations and added an additional 14,356 novel genes with 22,061 novel transcripts to create our new combined annotations (Figure 2A and Supplementary Figures 1A,B). We then refined these annotations using blastn. Including both novel transcripts and re-annotation of reference transcripts with cryptic gene symbols (e.g., c1orf1, LOC123, etc.), we were able to add gene symbols or a likely gene symbols for 5,093 genes and 7,336 transcripts. In sum, our new transcriptome assembly contains a total of 42,881 genes and 71,149 transcripts, with 96.3% of annotated genes located on the 17 longest contigs (Figure 2A), which approaches chromosome level contiguity for this female with 16 autosomes (Lemskaya et al., 2018). Based on the location of Xist and the large majority of regions syntenic (Grabherr et al., 2010) with the mouse X chromosome, Itri10 is the ground squirrel X chromosome (Supplementary Figure 1C).

### Differential Gene Expression in Hibernation

Next, we reanalyzed the strand-specific, paired-end RNA-seq data from 90 brain samples that were collected for a study of RNA editing in 13-lined ground squirrels (Riemondy et al., 2018, Supplementary Table 1). The dataset comprised five individuals from each of six states (Figure 2B) for three brain regions: forebrain, hypothalamus and medulla. We found 13,035 genes passed the count filter in at least one of these regions, with most (85%) detected in all three (Figure 2C). Genes were analyzed for differential expression by physiological state (Supplementary Datasheets 2–4 and Supplementary Tables 2–4). Three complementary but independent analytical approaches were used to interrogate this complex data set: random forest classification (Breiman, 2001), linear regression (Love et al., 2014), and weighted correlation network analysis (Langfelder and Horvath, 2008) followed by pattern clustering. The RNA-seq data from all three brain regions were also analyzed for state-specific changes in RNA structure caused by alternative splicing.

**FIGURE 3:**
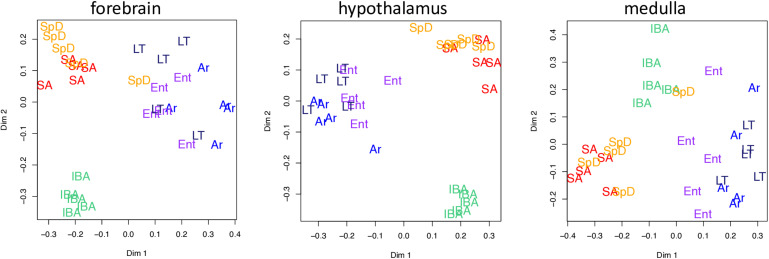
Two dimensional scaling plots following unsupervised random forest clustering of all pass-filter pseudocounts for each brain region as indicated. States are as defined in Figure 1 and individual animals are represented by their group name and color.

Unsupervised clustering of the gene-based relative abundance data from each brain region by random forests revealed that the transcriptome in IBA was the most distinct of these six physiological states in all three brain regions (Figure 3). The remaining hibernation groups, Ent, LT and Ar, were more closely juxtaposed. Animals from the two homeothermic groups, SpD and SA, also clustered closely together, but were generally well separated from the hibernators, including IBA despite its similarly warm Tb. These findings indicate that both seasonal and torpor-arousal cycles exert strong effects on the transcriptome in all three brain regions and reveal an extensive shift in the transcriptome within 2 h after Tb recovery during IBA compared to all other states.

**FIGURE 4:**
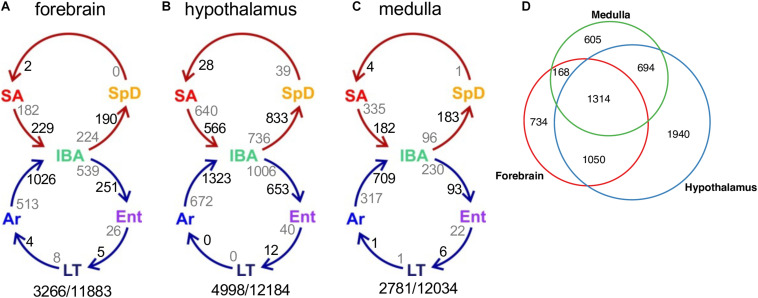
Differentially-expressed genes across circannual rhythm of hibernation in three brain regions. **(A–C)** Circles represent the homeothermic (red) and heterothermic (blue) portions of the hibernator’s year, arrows the sequence of physiological transitions. Two numbers are given on the inside of each arrow, decreasing in gray, increasing in black; their sum is the total DE between those two states (Supplementary Figure 2). Numbers outside the circles are the total number of DE genes (*q* < 0.001)/number of pass-filter genes detected in that brain region. **(D)** Venn diagram of common and unique DE genes among the three brain regions.

We next considered pairwise DE genes among the contiguous physiological states (Figures 4A–C and Supplementary Figure 2). Consistent with the random forest findings, a relatively large number of DE genes distinguished IBA from both states in the homeothermic group (SpD and SA) as well as from Ar and Ent hibernators in all three brain regions. The transcriptome was most altered as the hibernating animals transitioned out of torpor into the first 2 h of the short euthermic period (Ar to IBA), where a large increase of transcripts was observed. The transcriptome also changed substantially across the 12-h euthermic period of the interbout arousal (IBA to Ent), and between active and hibernation seasons (compare the IBA hibernator to either SpD or SA active animals). A far smaller fraction of the transcriptome was altered between Ent and LT, or especially LT and Ar, hibernators, or between the two active states, SpD and SA. Among the three brain regions, the hypothalamus exhibited the greatest proportion of DE genes, 41%, followed by forebrain and medulla with 27 and 23% DE, respectively. Significantly, despite the high qualitative similarity of the transcriptome across the three brain regions, there was far less quantitative similarity. We observed large differences in the DE genes among regions (Figure 4D) — just 20% were common to all three regions in contrast to 85% of all genes detected (Figure 2C). The enhanced proportion of DE genes in hypothalamus can only partially explain this skew. Rather the large shift in the proportion of DE genes common to all three brain regions suggests unique roles or responses of forebrain, hypothalamus and medulla in hibernation physiology, consistent with previous findings (Schwartz et al., 2013).

A closer inspection of the DE genes across the pairwise transitions most relevant to hibernation physiology provides additional insight (Supplementary Figure 2). The relative proportion of increased and decreased DE genes varied depending on the physiological states compared. Pairwise comparisons between IBA and the neighboring winter states of Ent or Ar reveal an excess of genes with increased relative transcript abundance in IBA in all three brain regions, indicating an elevated burst of transcription when Tb recovers after torpor. A more region-specific response was found in comparisons between IBA and either of the two, warm homeothermic states, SA and SpD. In forebrain, the number of genes increased in IBA slightly outnumbered those increased in either SA or SpD. In contrast, more genes were increased in SA and SpD than in IBA in hypothalamus (i.e., decreased for hibernation), and an even larger skew toward more genes with increased abundance in the two homeothermic states compared to IBA occurred in medulla. The maximum observed log2 fold change for any DE gene in any pairwise comparison varied from as low as −0.47 to as high as −5.15, but only a small fraction of genes (<5%) changed their relative abundance in the RNA pool by at least 2-fold (log2value ≥ 1 or ≤−1) and by far the largest number of these were seen in the comparison of IBA to Ar animals in all three brain regions (Supplementary Figure 2), a comparison that also included a large number of novel, i.e., unnamed (G#), genes.

**FIGURE 5:**
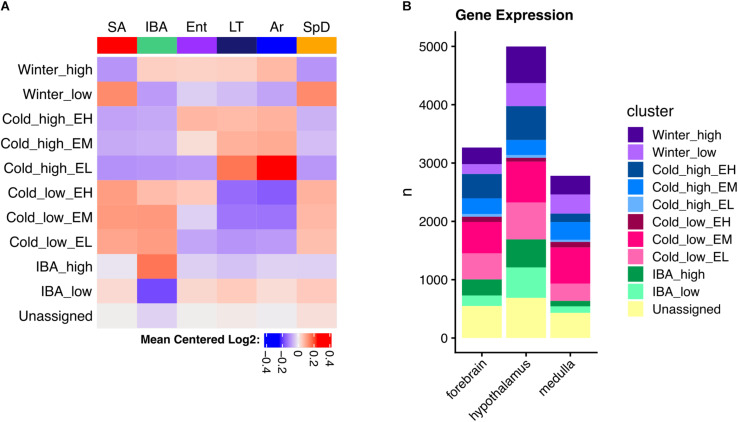
Clustered brain DE genes in hibernation. **(A)** Mean gene expression patterns for each cluster in hypothalamus; EH, EM, and EL indicate the relative abundance during entrance into torpor – high, medium or low, respectively. **(B)** Comparison of the number of genes in each cluster among three brain regions as indicated. See also Supplementary Figure 6.

The DE genes increased in IBA-stage hibernators are expected to include a mix of those driving and responding to both season and the torpor-arousal cycle. In an attempt to deconvolute these effects, we next applied weighted gene correlation network analysis (WGCNA, Langfelder and Horvath, 2008) to the full dataset for each brain region. This method identifies modules of genes with correlated relative RNA abundance patterns across all six physiological states. WGCNA can further correlate those modules with phenotypes, in this case a panel of attributes related to hibernation (Supplementary Table 1). Modules highly correlated (>0.8) to heterothermy, Tb and IBA were found in all three brain regions (Supplementary Figures 3–5), consistent with the random forest and linear regression results described above. Because we (and others, e.g., Botía et al., 2017) found WGCNA frequently failed to assign genes to the module where they were best correlated, we modeled specific patterns to capture the bulk of gene expression changes and reassigned DE genes to their best fit cluster (see section “Materials and Methods”). Just ten clusters captured 83, 86, and 84% of the DE genes in forebrain, hypothalamus and medulla, respectively (Figure 5A and Supplementary Figure 6). The cluster pattern with the greatest number of genes in each brain region was Cold_low_EM, i.e., decreased in the low Tb animals (LT and Ar) from intermediate levels during Ent (EM), whereas the fewest number of genes had the pattern, Cold_high_EL, i.e., were increased at low Tb despite having low relative abundance when animals entered torpor (EL), Figure 5B and Supplementary Figure 6.

As noted above, the DE genes were relatively brain region-specific among forebrain, hypothalamus and medulla (Figure 4D); thus, it is not unexpected that the genes found in analogous clusters were also largely distinct among the three brain regions (Supplementary Figure 6). Despite this apparent uniqueness, their functional enrichments were nonetheless similar or identical, revealing general patterns linked to phenotype (Supplementary Figures 7–9). Genes encoding proteins that bind polyA RNA and function in ubiquitin-mediated proteolysis were increased throughout the winter (Winter_high), contrasting with genes exhibiting the Winter_low pattern, which were biased in structural components including cell junction and extracellular matrix. All three of the patterns with increased relative abundance at low Tb (Cold_high_ with Ent either high, medium or low, i.e., EH, EM or EL) were enriched in terms related to mitochondrial structure and function, whereas those decreased at low Tb (Cold_low) were strongly enriched for proteins involved in transcription. Finally, polyA binding proteins were again enriched in the transcripts increased in IBA in all three brain regions whereas those decreased in IBA were related to axon or synaptic function and cell adhesion in forebrain and hypothalamus (Supplementary Figures 7–9 and Supplementary Table 5). Genes encoding proteins involved in mRNA translation/protein biosynthesis were also enriched in RNAs elevated at low Tb (Cold_high_) in both forebrain (EH) and hypothalamus (EM), but not medulla, which had substantially fewer DE genes than the other two brain regions and thus fewer significant enrichments were found.

### Genes Exhibiting High Fold-Change

The large number of DE genes revealed by this study precludes specific detailed discussion of each, although the custom data browser, squirrelBox, provides easy access to explore the full set of DE genes on both an individual and group (pairwise and clusters) basis. Here we illustrate some of squirrelBox’s capabilities by focusing on the small subset of genes with the largest fold changes (≥2-fold) between pairs of states (Supplementary Tables 2–4). A previous study of brown adipose tissue in these hibernators concluded that specific regulatory mechanisms are needed to account for ≥2-fold relative changes in transcript abundance, whereas intrinsic mRNA half-life differences can fully explain the majority of smaller fold-changes (Grabek et al., 2015). Because the high cell-type complexity in the brain (documented here for hypothalamus, Supplementary Table 6) likely means that regulated transcripts in small subsets of specialized cells are effectively diluted (Epperson et al., 2010), ≥2-fold DE changes in these brain regions are of particular interest.

**FIGURE 6:**
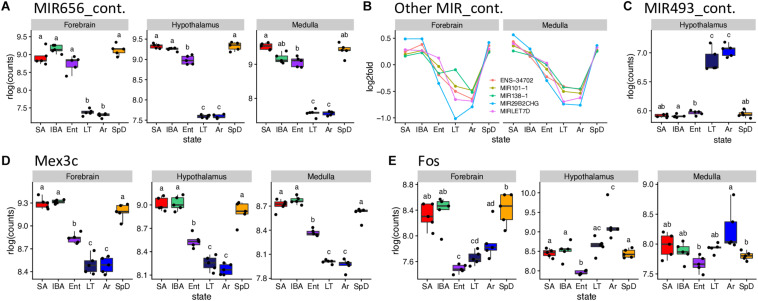
Selected DE genes with large fold changes in hibernation. RNA-seq pseudocounts for **(A)** MIR656_containing RNA from three brain regions. **(B)** Line plots of five other MIR_containing DE genes for forebrain and medulla: hypothalamus, not shown, is similar. **(C)** Boxplot of MIR493_containing miRNA precursor gene counts in hypothalamus. **(D,E)** Boxplots for the protein coding genes, Mex3C and Fos, respectively, in the three brain regions. For boxplots, dots represent log2 values for individual samples, boxes delineate the interquartile range, horizontal lines mark the group median, whiskers indicate the boundaries where sample dots lying outside are outliers, lowercase letters define different groups (*q* < 0.001). For line plots, dots represent the log2 fold change of mean value of expression for each state over overall mean expression. Full names for the genes in **(B)** are: ENSSTOG00000034702, MIR101-1_containing, MIR29B2CHG_containing_ G39119 and MIRLET7D_containing. See squirrelBox for additional information.

A set of miRNA-containing precursors that decreased 8 to 25-fold in LT and Ar compared to IBA in all three brain regions were among the highest fold-changes observed, including MIR656 (Figure 6A), MIR29B2CHG, MIRLET7D, MIR101-1, MIR138-1 and ENSSTOG00000034702 (Figure 6B). In stark contrast, another pair of miRNA precursors, ENSSTOG00000021167 and MIR493_containing (Figure 6C), increased dramatically (the highest in our dataset) during the cold stages, LT and Ar, particularly in hypothalamus. While their expression dynamics were generally smaller, several protein-coding genes were also ≥2-fold DE in all three brain regions, including: Chi3l1 (Winter_high), Unc13c (Winter_low), Dusp1 (Cold_high, note the relative abundance during entrance, EM, EL or EH, was variable among regions and is thus omitted), Clp1, Fadd, Kcnj2, Lig4, Lins1, Mex3c (Figure 6D), Pdk4, Slitrk6, and Znf646 (Cold_low; again, Ent variable), and Shisa2 (IBA_high).

Transcripts from additional genes that differed by at least 2-fold between at least one pair of states in one brain region included: Arc, Btg2, Fos (Figure 6E), Junb and Npas4 (Cold_low_EL), Entpd8 (IBA_high), and Piwil4 (Cold_high_EH) in forebrain; FosB, G21802, Txnip, Zfp36 (Cold_high_EL), Lysmd3 (Cold_low_EL), Pcdh18 (Cold_low_EM), Col11a1, Col19a1, Col2a1, Fzd2 (Winter_low) and Cga, Crym, Dio2, Hif3a (unassigned) in hypothalamus; and Hspa1a, Hspa1b (unassigned), B3gnt7 (Cold_high_EH), Tob2 (Cold_low_EL) and Ticam1 (Winter_low) in medulla. Taken together these genes provide a glimpse into the complexity of brain gene expression dynamics during hibernation.

### Factors Influencing RNA Stability

**FIGURE 7:**
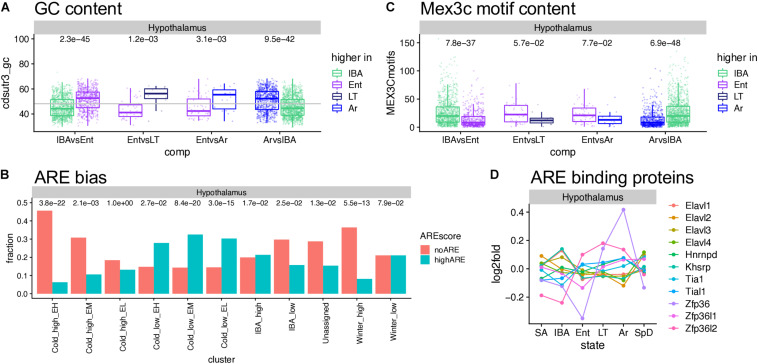
Sequence features affecting RNA stability and utilization. **(A)** GC content in hypothalamus varies across the torpor-arousal cycle; dots represent DE genes in the pairwise comparison indicated below, each assigned to the state where it increased; numbers above are the Wilcoxon test *p*-values. **(B)** The fraction of genes in each cluster that completely lack A-rich elements (ARE) compared to those with ARE scores of at least 8; numbers above are p values (Fisher exact test) comparing the number of genes in each category to the overall gene counts in that cluster. **(C)** Mex3c motif content varies across the torpor-arousal cycle, plot and numbers are as described for panel **(A)**. **(D)** Differential expression of mRNAs encoding ARE binding proteins. See Supplementary Figure 10.

The cohort of transcripts that increased in abundance across the torpor bout when *Q*10 effects virtually halt transcription (van Breukelen and Martin, 2002), present an interesting enigma. These relative abundance measurements reflect the balance between transcript synthesis and decay; in the absence of transcription, increased abundance must reflect increased stability. Our data provide numerous examples of transcripts that were stable or even increased over the prolonged period of low Tb (Cold_high_ EH, or EL and EM clusters, respectively, in Figure 5, Supplementary Figures 6–9, and Supplementary Tables 2–4), despite the profound depression of transcription at these temperatures. Moreover, we observed an enrichment of polyA RNA binding proteins in the IBA and Winter_high clusters. The presence or absence of *cis*-acting AU-rich elements (AREs, García-Mauriño et al., 2017; Otsuka et al., 2019), binding sites for other RNA binding proteins and miRNA target sites in 3′UTRs (Rissland, 2017), as well as the nucleotide composition of a transcript’s coding sequence and 3′UTR (Courel et al., 2019), all affect mRNA translation, storage and stability. Therefore, we analyzed the GC and ARE content of significantly up- or down-regulated genes. Among the three brain regions, the hypothalamus showed the clearest effect of GC content on the relative RNA abundance– the genes increased as Tb began to decrease and further increased as Tb remained low, i.e., increased in the second state in the pairwise comparisons IBAvsEnt, EntvsLT and EntvsAr, had significantly higher GC content than the genes whose relative abundance increased when Tb increased (IBA increased in the ArvsIBA comparison, Figure 7A). Medulla but not forebrain exhibited a similar pattern (Supplementary Figure 10A), and not surprisingly, the ARE scores were reciprocal to the GC content (Supplementary Figure 10B). Comparing transcripts with no AREs to those with high ARE scores revealed a strong bias of transcripts lacking AREs in Cold_high_EH and Winter_high clusters. In contrast, transcripts with high ARE scores were enriched in the Cold_low_EL and Cold_low_EM clusters (Figure 7B and Supplementary Figure 10C). Mex3c, another RNA binding protein involved in post-transcriptional regulation of its target RNAs, is one of the strongest DE protein coding genes in our dataset (Figure 6D) and a member of the Cold_low_EM cluster across all three brain regions. In contrast to the ARE binding proteins, the mechanism of action of Mex3c has not yet been revealed in detail; nevertheless, it is known to be involved in a number of physiological processes, including energy expenditure (Jiao et al., 2012), and a recognition sequence has been described (Yang et al., 2017). We again find Mex3c motifs enriched in transcripts elevated in IBA compared to other hibernation states (Figure 7C and Supplementary Figure 10D). Finally, mRNAs that encode several ARE binding proteins are also DE in hibernation; these were most dynamic in hypothalamus and cycle asynchronously (Figure 7D and Supplementary Figure 10E). Taken together, these data are consistent with a complex and dynamic regulation of RNA stability during torpor-arousal cycles of hibernation.

### Alternative Splicing in Hibernation

**FIGURE 8:**
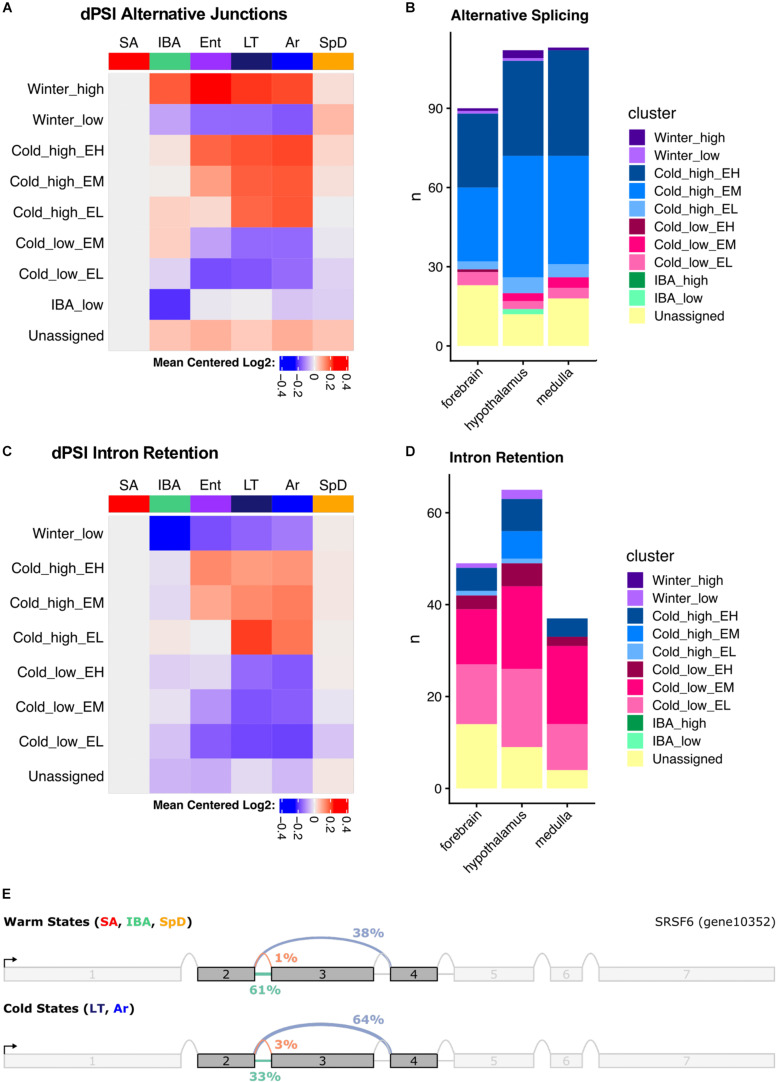
Summary of alternative splicing in the hypothalamus. **(A)** Mean dPSI patterns relative to SA and **(B)** summary of clusters for alternative junctions. **(C)** Mean dPSI patterns relative to SA and **(D)** summary of clusters for retained introns. In **(B,D)**, brain regions are indicated below each bar, n is the number of genes, colors differentiate clusters. **(E)** SRSF6 splice graph from MAJIQ analysis illustrates temperature-dependent alternative splicing.

We used MAJIQ (Vaquero-Garcia et al., 2016) to identify alternative splicing events, both to improve transcript annotation (see section “Materials and Methods,” Figure 2B) and to explore state-specific splicing changes. MAJIQ models alternative splicing in terms of local splicing variations (LSVs). A MAJIQ LSV consists of all possible junctions observed for a given source (upstream) or target (downstream) sequence. MAJIQ quantifies the relative abundance (percent spliced in, PSI) of all junctions in an LSV and then identifies significant changes in relative abundance (dPSI) between pairs of samples. We ran MAJIQ on all pairwise state comparisons, and clustered significant LSVs (*q* ≤ 0.001) into the 10 most commonly observed patterns, as described above for the gene expression data. We found that alternative splicing, defined here as a significant increase in the PSI of all alternative junctions relative to the “summer-dominant junction” (most common junction in SA), is largely temperature dependent and most prevalent in Cold_high_EH and EM genes (Figures 8A,B). Intron retention, in particular, is highly temperature dependent and most prevalent in Cold_low_EM and EL genes (Figures 8C,D). However, rather than being ‘alternative’ splicing events as defined above, intron retention is the default homeothermic state at most significant LSVs that involve a retained intron (i.e., the retained intron has the highest PSI in SA). We observed multiple common classes of temperature-dependent alternative splicing events, including the aforementioned intron retention in warm states at the alternative splicing-related SRSF6 locus (Figure 8E and Supplementary Figure 11A), exon skipping in the cold states at the mTORC1-regulating Pip4k2c locus (Supplementary Figure 11B), and the use of alternative 5′ splice sites at the brain development-related Cttnbp2 locus (Supplementary Figure 11C). In addition to temperature-dependent events, we also captured smaller numbers of seasonal and IBA-specific events. These included a complicated event at the ACSM1_like locus in which intron retention in the summer and spring is replaced with exon skipping in the winter (Supplementary Figure 11D), and IBA-specific intron retention at the Sec31a locus (Supplementary Figure 11E). Notably, these examples are all in the coding sequence of the transcript and predict an altered protein product.

The three brain regions shared a pattern of increased alternative splicing in the cold, but very few significant LSVs were common among regions (Supplementary Figure 12). Interestingly, the greatest overlap occurred in the large Cold_high_EH cluster, which most aggressively segregated the warm and cold states (23–32% of the significant LSVs in each region were common to all three brain regions in this cluster). This is in stark contrast to the other large cluster, Cold_high_EM, where only 4–7% of the significant LSVs were common across regions. LSVs in individual genes in each brain region are plotted in Supplementary Figures 13–15.

We also observed that intron retention events were common among the significant LSVs identified by MAJIQ. Figure 8D summarizes the state-dependent patterns of intron retention in these samples, demonstrating a consistent decrease of retained introns in the cold states. This is notably inverted relative to the alternative splicing patterns and demonstrates that intron retention is the default state of these LSVs when the animals are warm. This common cold-specific excision of introns may be the result of suboptimal *cis*-acting splicing sequences being recognized more frequently by the splicing machinery in the cold states than the warm states, as the splice sites flanking introns excised in the cold are statistically significantly weaker than those flanking all non-overlapping, non-significant introns (Supplementary Figure 16). As in the general alternative splicing analysis, the most significant overlap between the brain regions occurred in the cluster that most aggressively segregates the warm and cold states, “Cold_low_EL” (Supplementary Figure 17). In this cluster, 24–40% of the significant intron retention events in each region were shared among regions. Individual genes with intron retention events in each brain region are plotted in Supplementary Figures 18–20.

### Comparison to Earlier Work

Very few datasets that examine differential gene expression in hibernator brain using high-throughput screening methodology are available for comparison to these data. A 2013 study of hypothalamus and cortex, also in 13-lined ground squirrels (Schwartz et al., 2013) is the most comparable. RNA-seq data were collected from three biological replicates (each a pool of one male and one female) in four physiological groups: non-hibernators in October and April and mid-season hibernators during torpor and IBA. We re-filtered our hypothalamus and forebrain data to include all DE genes with pairwise differences (padj < 0.05) between IBA and SpD, SpD and SA, SA and IBA, and IBA and LT, to allow direct comparison to the pairwise differences reported between IBA and April, April and October, October and IBA, and IBA and torpor (Schwartz et al., 2013), respectively. The gene count distributions were similar between the two datasets (Supplementary Figure 21, top panels), but the increased read depth in our study uncovered many additional DE genes. In hypothalamus, approximately half of the DE genes in Schwartz et al. (2013) were recaptured in the present dataset, and the common DE genes were well correlated. The comparison of forebrain to cortex also showed strong correlation of DE genes from the two datasets, although these were fewer, likely reflecting anatomical differences between the tissues used for RNA isolation. The largest fold change in relative transcript abundance (increased throughout winter hibernation) that was common to the two datasets in both brain regions (bottom panels, Supplementary Figure 21) was Chi3L1.

## Discussion

The goal of this study was to characterize gene expression variation across the spectrum of the hibernator’s extraordinary physiology. We hypothesized that genes involved in tissue protection and metabolic suppression during hibernation are differentially expressed by timing in the seasonal or body temperature cycle, respectively, and thus would be revealed by the relative abundance of their transcripts in our sample groups. While ours is not the first study with this goal (Schwartz et al., 2013; Lei et al., 2014; Nespolo et al., 2018), it uses a more precisely defined and larger set of samples, as well as an improved genome and annotation, to separate for the first time seasonal from torpor-arousal cycle effects on the transcriptome in forebrain, hypothalamus and medulla. We document that both season and body temperature have large quantitative (Figures 3–5 and Supplementary Figures 2–9) and smaller qualitative effects on the transcriptome (Figure 8 and Supplementary Figures 12–15, 17–20) in all three brain regions examined.

The vast majority of transcripts were detected in all three brain regions (Figure 2C), yet transcriptome dynamics during circannual hibernation were substantially more region specific (Figure 4D). This view is supported by results of both the pairwise (Figure 4D) and cluster (Supplementary Figure 6) based analyses and is consistent with earlier findings in hypothalamus and cortex from the same species (Schwartz et al., 2013). Some of the brain region-specific differences are likely due to bona fide functional differences. For example, the critical role of the hypothalamus in reproduction, feeding behavior, metabolism and thermoregulation, all variable across the timepoints studied, likely contribute to the large number of DE genes compared to medulla and cortex. Moreover, the hypothalamus remains active throughout the torpor-arousal cycle compared to the neocortex (a substantial component of our forebrain region) which is the first region to become inactive during entrance into torpor and the last to be reactivated upon arousal (Sonntag and Arendt, 2019). Differences among brain regions in neuronal activity as measured by 2-deoxyglucose uptake, blood flow dynamics and c-fos gene expression are well documented in hibernators (Kilduff et al., 1990; Frerichs et al., 1994; Bratincsák et al., 2007). But a closer inspection of many individual genes using squirrelBox suggests that these analyses also amplify subtle quantitative differences in transcripts that are qualitatively more similar among regions. Where the same transcript reaches our expression limit for detection in all three brain regions, an exaggeration of regional differences is often attributable to smaller fold changes and higher within group variability, e.g., Wnk4, a [Cl^–^]-sensitive kinase that regulates cation/Cl^–^ cotransporters (Tang, 2016), is clustered with Winter_low in forebrain, IBA_low in hypothalamus and unassigned in medulla (squirrelBox). Yet the transcript varies similarly across the six physiological groups with different within group variability among brain regions. The brain’s cell-type complexity likely also contributes to variability in our data, e.g., small variations in macroscopic dissection of hypothalamus and medulla, or subsampling of the pulverized forebrain could alter the cell composition of the tissue and thus the relative abundance of transcripts that are highly cell-type specific in the extracted RNA population, introducing variability.

**FIGURE 9:**
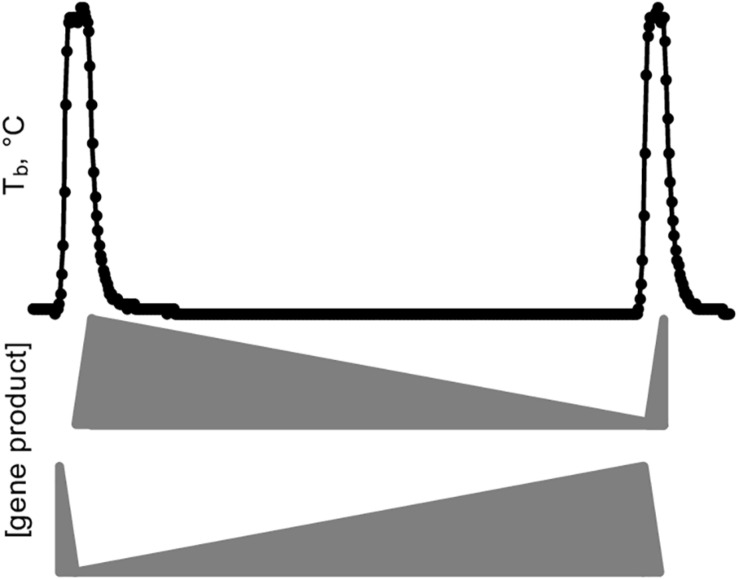
Simplified model of gene expression dynamics during a torpor-arousal cycle in hibernation. Body temperature (Tb) trace plots two IBAs flanking one torpor bout. Middle, the relative abundance of a hypothetical “hibernation gene” that is transcribed during IBA and degraded during torpor, eventually reaching a threshold value that triggers arousal, where it can be replenished and start the cycle anew. Meanwhile a different gene product (bottom) protected from degradation and hence accumulates during torpor initiates the synchronized cascade of gene expression that ultimately leads to the next torpor bout, including correct timing for synthesis of the hibernation gene.

A simple schematic illustrates the crucial importance of sample collection in capturing DE genes during hibernation (Figure 9). A hypothetical gene product critical for torpor is synthesized during IBA, but, in the absence of transcription at low Tb (van Breukelen and Martin, 2002), slowly degrades during the torpor bout. Alternatively, a gene product important for arousal is stabilized during torpor and degrades during IBA. It is clear from these examples that sampling several torpid or aroused hibernators without precise knowledge of their timing would average to the same relative abundance and thus leave both types of DE genes undiscovered.

The simplified example in Figure 9 falls far short of explaining the gene expression dynamics of hibernation as captured in our dataset, however, which reveals a complex integration of transcription and RNA turnover rates. The importance of regulating both is broadly reflected by the cluster gene enrichments showing unique differential expression of positive and negative transcription factors and RNA binding proteins. While RNA-seq measures steady-state RNA and not transcription, the profound temperature sensitivity of the transcription machinery (van Breukelen and Martin, 2002) likely means transcripts that maintained their abundance across the entire torpor bout (Winter_high, Cold_high_EH, IBA_low) have average stability compared to the overall transcript pool. In contrast, RNAs that decreased across the torpor bout, i.e., in the clusters Cold_low_EH and EM, are particularly unstable. Finally, the small group of RNAs that actually increased in the absence of transcription (Cold_high_EH and EM), are likely specifically stabilized, newly polyadenylated during torpor, or a combination of the two (Grabek et al., 2015), although we cannot rule out the possibility that very low rates of transcription at the low Tb of torpor can account for the increase in a few transcripts.

Fos provides a noteworthy example of a transcript that increased across the torpor bout, i.e., from Ent to LT to Ar in all three brain regions (Figure 6E). Its pattern is unassigned in hypothalamus and medulla but is Cold_low_EL in forebrain. This pattern suggests that Fos mRNA is particularly well-stabilized across the torpor bout or possibly even transcribed in limited cell types, with the latter view consistent with in previous situ hybridization results in hypothalamus (Bratincsák et al., 2007). Its further increase in Ar may reflect the onset of new transcription as Tb recovers, with the brain warming significantly faster during Ar (MacCannell et al., 2019) than the animal’s abdominally measured Tb. This view is supported by the outlier arousing ground squirrel in all three brain regions whose Tb is significantly higher than that of the other Ar animals (#53 with Tb = 12.8 vs. 7.9 ± 0.5°C, squirrelBox), suggesting it is farther along in the process of arousing from torpor than the other Ar individuals studied here. Other immediate early genes with kinetics similar to Fos are the transcription factors Jun and Junb, and Zfp36, which destabilizes transcripts containing AREs, including itself. The rapid increase and then decrease of these transcripts indicate that additional precisely-collected timepoints later during the rewarming process and during the IBA are needed to fully capture the transcriptional program of the torpor-arousal cycle.

It is reasonable to postulate that the genes exhibiting larger fold changes reflect specific regulation because of their involvement in the hibernation phenotype rather than simple, intrinsic variation of RNA stability. In our dataset, relatively few of the 6,505 DE genes changed by greater than 2-fold, likely because the high cell-type complexity in the brain masks larger fold changes that occur in smaller subsets of cells (Epperson et al., 2010). The largest fold changes observed were in a set of miRNA precursor genes common to all three brain regions (Figures 6A,B). These decreased dramatically when the animals were at low Tb (Cold_low_EM or_EH). Further work is needed to determine whether their loss reflects processing and increased abundance of their embedded miRNAs and to identify the regulatory targets of those miRNAs. Interestingly two other miRNA precursors accumulated (Figure 6C, and ENSSTOG000000021167 in hypothalamus and medulla) equally dramatically in the cold, consistent with these high fold changes reflecting regulated processes rather than a general uncoupling of synthesis and processing at low temperature, as shown previously to occur during hibernation torpor for transcripts degraded by the nuclear exosome (Grabek et al., 2015).

Changes in the relative abundance of transcripts of known protein coding genes were of smaller magnitude than these miRNA precursors, but several with ≥2-fold change have plausible roles in hibernation. Two seasonally DE genes in all three brain regions have known functions that would contribute to the hibernator’s enhanced neuroprotection. Chi3L1, increased throughout winter (Supplementary Figures 21B, C), induces oligodendrogenesis after inflammatory damage (Starossom et al., 2019) and Unc13c, decreased throughout winter, lowers synaptic activity (Palfreyman and Jorgensen, 2017). In torpor arousal cycles, the inwardly rectifying potassium channel, Kcnj2, is decreased in the cold. This channel modifies the excitability of neurons (Trimmer, 2015) and drives vasodilation to alter regional blood flow (Longden et al., 2017), a feature of hibernation (Frerichs et al., 1994). Other genes that altered by at least 2-fold in one or two of these brain regions may also contribute to the hibernators enhanced neuroprotection. Three non-fibrillar collagens decreased throughout winter in hypothalamus, Col11a1, Col2a1, and Col19a1 (and the first two were also altered similarly in medulla); these localize to the extracellular matrix and may facilitate synapse formation (Su et al., 2010). In forebrain, Arc and Npas4 decreased in the cold with the pattern Cold_low_EL, Arc mediates synaptic plasticity (Barylko et al., 2018) and may help reestablish synapses that retract during torpor but re-form, apparently early, during IBA (von der Ohe et al., 2006). Npas4 is neuroprotective after ischemia (Choy et al., 2015) which occurs transiently with minimal damage each time the hibernator arouses from torpor (Dave et al., 2012). In hypothalamus, Fzd2 which decreased in winter, attenuates pathological Ca^++^ increase in a rat model of traumatic brain injury (Niu et al., 2012). Also in hypothalamus, accumulation of Txnip1 during torpor could beneficially signal redox stress during arousal, with its quick drop again in IBA blunting its potential for damaging effects (Nasoohi et al., 2018), and the drop of Hif3a in IBA could help assure a robust transcriptional response to hypoxia. In medulla, the winter decreased Ticam1 reduces microglia activation, lowers proinflammatory cytokines and enhances neuron survival (Wang et al., 2018). Id2 is lowest in IBA; when this gene is silenced, hypoxia/ischemia-induced neuronal injury is attenuated because neuronal apoptosis is inhibited (Guo et al., 2015).

The transcripts of several genes linked to functions other than neuroprotection were also highly DE in our dataset. Dusp1, which negatively regulates of cell proliferation and responds to cellular stress, was dynamic across the torpor-arousal cycle, increasing during torpor to 2× higher in Ar than IBA in hypothalamus and medulla. Tob2 and Btg2, both antiproliferative (Ezzeddine et al., 2012; Micheli et al., 2015), were decreased in torpor (Cold_low_EL or EM) in all three brain regions, as was Lig4, a DNA repair enzyme. Fadd, initially found to function as an adaptor molecule for death receptor-mediated apoptosis, but now known to be involved in non-apoptotic cellular processes including proliferation and cell-cycle control as well as regulation of gene expression and control of metabolic pathways (Marín-Rubio et al., 2019), likewise exhibits decreased expression in the cold. PiwiL4 increased from IBA to Ent in all three regions, it is an RNA mediated transcriptional repressor, consistent with the generalized repression of transcription during the torpor bout. Among the more region-specific genes Entpd8 is a diphosphatase that alters extracellular nucleotide abundance (Fausther et al., 2007), including of the hibernation-relevant adenosine (Jinka et al., 2011), which is elevated in medulla during IBA. Three genes related to thyroid hormone action are ≥2fold increased in the hypothalamus of SpD compared to SA animals: Cga, Crym and Dio2. Thyroid hormone action is important for both reproduction and metabolism – high reproductive hormones are incompatible with hibernation (Darrow et al., 1988; Richter et al., 2017), and there is a clear depression of metabolic rate prior to the onset of heterothermy (Concannon et al., 1999; Sheriff et al., 2012). Because the relative abundances of Cga and Crym transcripts in the reproductively active SpD animals were indistinguishable from the reproductively quiescent hibernators, these genes are likely more important for the metabolic effects of thyroid hormone in the hypothalamus. In contrast, the low levels of Dio2 in the 20 hibernators were more similar to the SA animals, suggesting it is more important for reproduction, a view bolstered by the outlier male ground squirrel in IBA whose tissue was collected in late March (#78, squirrelBox), at least one full month later in the year than all of the other hibernators in this study. Elevated protein chaperones Hspa1a and Hspa1b in medulla during SA may reflect a seasonal remodeling of electrophysiological properties to protect against respiratory failure at low temperatures, as shown for hamsters prior to hibernation (Russell et al., 2019). Of course, for all of these protein-coding genes, their biological activity will further depend on the synthesis, stability and post-translational modifications of their corresponding proteins.

In addition to previously annotated genes, there are multiple transcripts that remain unannotated that are also ≥2-fold DE in our dataset and hence may reflect regulated processes of particular interest to understanding mechanisms important for hibernation. Some of these, e.g., *G21802* in hypothalamus, likely reflect accumulation of intermediates that would not be seen but for differential effects on RNA synthesis and processing caused by the prolonged time at low Tb. G21802 is a U2 snoRNA that is processed from an intron in a protein coding gene, *Cep295*, which was not differentially expressed. Others of these, however, likely warrant further investigation, including G14950, which is decreased at low Tb in all three brain regions (Cold_low_EM) and is predicted to host a micropeptide (squirrelBox). Interestingly, this gene lacks homology to transcripts in human or mouse, despite sequence similarity to, and hence the presence, of the likely corresponding chromosomal region in the human genome, which is peppered by transposable element insertions. If the ability to hibernate is ancestral to mammals but lost among species that no longer hibernate (Srere et al., 1992), the remnants of the critical genes should be present to varying extents in non-hibernators including humans and mice, due to the relaxation of selective pressure for function. However, if a homologous, intact gene is found in other hibernators, it would be particularly interesting for follow-up functional analyses.

The transcriptome dynamics of the individual genes discussed above likely reflect molecular mechanisms controlling both RNA transcription and turnover. A relatively synchronized burst of transcription occurs as Tb recovers during arousal, and then the transcriptome changes dramatically over the 12-h euthermic period and again over the much longer period of torpor. Suppressed translation at low Tb (van Breukelen and Martin, 2001) would protect GC-rich, optimally translated mRNAs from their normal rates of degradation (Courel et al., 2019), allowing their increase across the torpor bout. The enrichment of RNA binding proteins among the DE genes uncovered by this study, and their complex abundance dynamics reveal a rich regulatory network that controls RNA stability seasonally and during the torpor-arousal cycle. Although ARE elements and their binding proteins were initially discovered for their role in promoting rapid turnover of transcripts that require tight regulation, i.e., genes with roles in cell proliferation or immune response, they are now known to operate with far more finesse. Some ARE binding proteins stabilize transcripts rather than cause their degradation, and signaling via post-translational modification may specifically stabilize or destabilize a given transcript (García-Mauriño et al., 2017; Otsuka et al., 2019). Taken together, the observations support a role for both transcriptional and post-transcriptional mechanisms in sculpting transcriptome dynamics during hibernation.

We also provide evidence for hibernation-associated RNA structural variation. Modeling the exact effect of the observed differential splicing on the corresponding protein is difficult because it is impossible to faithfully reconstruct mRNA isoforms from short-read sequencing at complex loci (Vaquero-Garcia et al., 2016). Nonetheless, two of these loci, *SRSF6* and *Pip4k2c*, are simple enough to make reasonable predictions about the proteins produced by alternative splicing.

In the cold states, the SRSF6 protein is 342 amino acids long and contains two highly conserved N-terminal RNA recognition motifs (RRM1 and RRM2). In the warm states, retention of the second intron (Figure 8E) results in a frameshift that truncates the protein to 192 amino acids and removes the RRM2 domain. Recent evidence shows that the RRM2 domain is required for SRSF6 to regulate alternative splicing (Wan et al., 2019), suggesting that temperature-dependent splicing of SRSF6 itself may be directly involved in the increase in alternative splicing observed in the cold states. Similar intron retention events that remove the RRM2 domain in SRSF6 are observed in several species, including *Drosophila melanogaster* and *Caenorhabditis elegans*, but not in humans (Shao et al., 2012). A similar cold-specific intron excision event is also observed at the closely related Srsf5 locus in all three brain regions (Supplementary Figures 18–20). Retention of this Srsf5 intron in the warm states would also eliminate the RRM2 domain and introduce a premature stop codon. The temperature-dependence of these two events is particularly interesting, as unproductive splicing events leading to non-sense-mediated decay (NMD) is a highly conserved regulatory mechanism employed by SR-family transcripts across evolution (Lareau and Brenner, 2015).

The result of the exon skipping event in Pip4k2c (Supplementary Figure 11B) is much more subtle than the SRSF6 intron retention event; there is an in-frame deletion of 13 amino acids in the cold states relative to the warm states. This deletion occurs in the middle of the only known functional domain in the protein, a phosphatidylinositol phosphate kinase (PIPK) domain, but the effect on kinase activity is unknown. The PIPK domain is identified in both isoforms, albeit with a lower score in the shorter isoform. However, if the cold-dominant shorter isoform does have altered kinase activity it may play an important role in hibernation, as Pip4k2c modulates mTORC1 activity (Shim et al., 2016) and mTORC1-dependent translational control is known to play multiple roles in brain development and function (Santini and Klann, 2011).

Alternative splicing may also play an important role in controlling gene expression dynamics during hibernation. The majority of the alternative splicing events in our data are clearly temperature dependent, and thus may reflect a passive response rather than a regulated process. The data suggest that lower affinity splice sites are being recognized more efficiently by the spliceosome in the cold. This may simply reflect slowed molecular activity, including dramatically slowed transcription at lower temperatures; transcription rates are known to affect splicing (Saldi et al., 2016). Nonetheless, as discussed above at least some of the observed alternative splicing events, including cold-dependent intron excision, would alter the coding sequence of proteins plausibly connected to hibernation. Robust cold state-specific intron-excision at the Srsf5 and SRSF6 loci, in particular, may be directly involved in modulating other alternative splicing events. Unfortunately, the use of short-read RNA-seq limits our ability to reconstruct transcript isoforms, especially those from complex loci. The per-junction events that we do observe, however, strongly suggest that long-read RNA-sequencing is likely to reveal substantial transcript isoform diversity as a function of hibernation physiology.

This analysis provides one early step toward unraveling the complex gene expression dynamics that orchestrate and enable hibernation’s extreme physiology. Importantly, these data demonstrate the utility and necessity of precisely timed samples for such work. The success of future experiments to discover key hibernation genes by exploiting their differential expression in the brain requires additional carefully collected samples to capture the complex dynamics of gene expression changes that occur later in the process of arousal, and at several timepoints across the IBA. Single-nucleus RNA-seq would help overcome problems caused by the high cell-type complexity in the brain. Nevertheless, the robust DE genes revealed in this study provide a rich set of gene-based hypotheses for guiding future work. The strongly DE, but presently uncharacterized genes, whether they likely encode proteins, lncRNAs or miRNAs, are of particular interest for future work that could lead to hibernation-based strategies for tissue protection or reversible metabolic depression.

## Materials and Methods

### Genome Enhancement

The existing 2011 SpeTri2.0 genome was further assembled using HiC (Dovetail genomics), resulting in a substantially improved genome. For this, a Chicago library was prepared as described (Putnam et al., 2016). Briefly, ∼500 ng of HMW gDNA (mean fragment length ≥50 kbp) were reconstituted into chromatin *in vitro* and fixed with formaldehyde. Fixed chromatin was digested with *Dpn*II, the 5′ overhangs filled in with biotinylated nucleotides and free blunt ends were ligated. After ligation, crosslinks were reversed and protein was removed, as was biotin that was not internal to ligated fragments. The purified DNA was then sheared to ∼350 bp mean fragment size and sequencing libraries were generated using NEBNext Ultra enzymes and Illumina-compatible adapters. Biotin-containing fragments were isolated using streptavidin beads before PCR enrichment of each library. The libraries were sequenced on an Illumina HiSeq 2500 (rapid run mode) to produce 150 million 2 × 101bp paired-end reads, which provided 52.6× physical coverage of the genome (1–50 kb pairs). Three Dovetail HiC libraries were prepared in a similar manner as described previously (Lieberman-Aiden et al., 2009). Briefly, for each library, chromatin was fixed in place with formaldehyde in the nucleus and then extracted. Fixed chromatin was digested with *Dpn*II, the 5′ overhangs filled in with biotinylated nucleotides, and then free blunt ends were ligated. After ligation, crosslinks were reversed, and the DNA purified from protein. Purified DNA was treated to remove biotin that was not internal to ligated fragments. The DNA was then sheared to ∼350 bp mean fragment size and sequencing libraries were generated using NEBNext Ultra enzymes and Illumina-compatible adapters. Biotin-containing fragments were isolated using streptavidin beads before PCR enrichment of each library. The libraries were sequenced on an Illumina HiSeq X. The number and length of read pairs produced for each library was: 130 million, 2 × 150 bp for library 1; 175 million, 2 × 150 bp for library 2; 211 million, 2 × 150 bp for library 3. Together, these Dovetail HiC library reads provided 8,356.58× physical coverage of the genome (10–10,000 kb pairs). Finally, the *de novo* assembly (SpeTri2.0), Chicago library reads, and Dovetail HiC library reads were used as input data for HiRise, a software pipeline designed specifically for using proximity ligation data to scaffold genome assemblies (Putnam et al., 2016). An iterative analysis was conducted. First, Chicago library sequences were aligned to the draft input assembly using a modified SNAP read mapper^1^. The separations of Chicago read pairs mapped within draft scaffolds were analyzed by HiRise to produce a likelihood model for genomic distance between read pairs, and the model was used to identify and break putative mis-joins, to score prospective joins, and make joins above a threshold. After aligning and scaffolding Chicago data, Dovetail HiC library sequences were aligned and scaffolded following the same method. We named this genome HiC_Itri_2, reflecting the assembly method and that this is the second super-assembly of SpeTri2.0 (Grabek et al., 2019).

### RNA Isolation and RNA-Seq

RNA was isolated from frozen (−80°C) brain regions dissected as described in Supplementary Datasheet 1 from five individuals (Hindle and Martin, 2013) representing each of six, precisely defined hibernation states based on time of year and Tb measured using implanted telemeter or iButton, or rectal probe, at time of tissue collection (Figure 1 and Supplementary Table 1): SA, summer active (08 August); IBA, interbout aroused (17 December–26 March, 2–3 h after Tb reached 30°C following >5 days torpid with Tb < 6.5°C, all with Tb 30.6–37.4°C); Ent, entrance (03 January–31 January, 23–27°C after 10–14 h with Tb > 30°C); LT, late in a torpor bout (31 January–26 February, Tb 5.5–7.2°C, with near constant, low Tb for 80–95% of the length of the previous torpor bout); Ar, arousing from torpor (28 December–22 January, with Tb spontaneously increasing to 7–12.8°C after multiple days in hibernation with near constant, low Tb); SpD, spring dark (21 March–30 April, after at least 10 consecutive days with Tb 35.6–37°C). Strand-specific RNA-sequencing libraries were prepared (TruSeq Stranded mRNA Library Prep Kit, Illumina) and sequenced as described (Riemondy et al., 2018). The raw read data were deposited at GEO (accession number GSE106947), after analysis for RNA editing (Riemondy et al., 2018), but here are analyzed for differential gene expression and isoform variation across hibernation states for the first time.

### HiC Genome Annotation

First, we leveraged our extensive RNA-seq data to create a custom annotation of the new genome assembly, HiC_Itri_2. For this, three independent annotations: (1) the NCBI Release 102 annotation; (2) Ensembl 90 annotation; and (3) a merge of all confidently expressed regions of the genome based on our RNA-seq data, were merged stepwise onto the HiC assembly. The last comprised strand-specific, paired-end RNA-seq data collected from the 90 brain samples in the present study, plus samples from neonate and testes as described (Riemondy et al., 2018) and an additional 25 liver, 9 adrenal and 9 kidney samples to further enhance transcript discovery and annotation. Trimmed, high quality RNA-seq reads from all 138 samples were first mapped to the 13-lined ground squirrel mitochondrial genome (Supplementary Table 1). The remaining non-mitochondrial reads (92.2 ± 2.3%) were then mapped to the HiC genome using HISAT2 (Pertea et al., 2016). Next, the deduplicated, uniquely mapping reads from each sample were assembled into a transcriptome with StringTie [-j 3 –c3, -rf, (Pertea et al., 2015)], these were merged across all samples and annotated (NCBI annotation release 102) using TACO (Niknafs et al., 2017), as described (Riemondy et al., 2018).

Comparison of the three annotations revealed that the NCBI annotation often better reflected the transcripts detected in our merged RNA-seq based annotation than the Ensembl annotation. This is likely because the NCBI annotation includes cDNA and RNA-seq data deposited in GenBank together with Ensembl’s homology-based annotation. But many loci in the Ensembl (Release 90) annotation, particularly small RNAs, were absent from the NCBI annotation. Because these were also detected in our ground squirrel RNA-seq data, all Ensembl transcripts that had no exon overlap with those from NCBI as well as short (<1 kb), single-exon Ensembl transcripts that had <90% reciprocal overlap with any NCBI transcripts were added (to retain the ability to capture primary transcripts of processed short RNAs). Finally, we added any remaining novel annotations from our merged RNA-seq data to the new Ensembl-supplemented NCBI annotations using the same rules as above.

We next used MAJIQ (v2.0, Vaquero-Garcia et al., 2016) in a novel application to complete the annotation and better integrate the previously unannotated transcripts represented in the data, which included fragments of retained introns, read-through events, and novel exons, with the reference annotations. After running MAJIQ Builder on the combined annotations (NCBI + Ensembl + novel transcripts), we extracted exon and intron retention information from the MAJIQ splicegraph and used these data to reassign or remove many novel transcripts and genes. In this step, the novel transcripts with evidence for splicing to a reference transcript (≥1 spliced read required) were re-assigned the gene ID of the reference transcript, and novel transcripts without evidence of splicing to a reference transcript and contained within a retained intron were removed from the annotations. Finally, we attempted to assign gene symbols to novel transcripts based on homology to human or squirrel RefSeq annotations. For this, we took all blastn results against the human RefSeq database with *e* ≤ 1e-20 and assigned the most significant gene symbol to each query transcript. The suffix “_like” was appended to the gene symbol if the query coverage was less than 90%, but higher than 50%, and the “_containing” suffix was appended if the coverage was less than 50%. Query transcripts with no blastn matches (e ≤ 1e-20) in human RefSeq were blasted against the *I. tridecemlineatus* RefSeq database (e ≤ 1e-20), which contains many predicted transcripts with no genome location information. Gene symbols were assigned as described above for the human blastn matches. See Supplementary Figure 22.

### smORF Annotation

Sequences from all annotated transcripts were processed via the micropeptide prediction tool MiPepid. Reported short open reading frames of length ≥10 aa, in the MiPepid classification class of “coding,” and probability ≥0.9 were labeled as “MiPepid_predicted.” The similarities of these sequences to high confidence smORFs documented in the SmProt database were then calculated using blastp-short parameters for sequences of length ≤30 aa and default parameters on longer sequences. Homologous smORFs were annotated with the SmProt micropeptide when blastp results escore ≤ e−10, percentage identical ≥90, and alignment coverage of the subject sequence ≥0.9. These analyses are provided as columns in the squirrelBox gene summary table for exploration.

### Transcript and DE Quantification

After demultiplexing, the reads were trimmed with cutadapt (Martin, 2011) to remove adaptor sequences and low-quality bases (*Q* < 10). All reads that were at least 20 nt after trimming were assigned to transcripts in the newly constructed *I. tridecemlineatus* transcriptome described above using Salmon (Patro et al., 2017) with –numBootstraps 50 (Additional File 7). The Salmon assignments were imported into R (Team, 2019) and summarized at the gene level using tximport (Soneson et al., 2015). We filtered the data, retaining only those genes in each brain region where rlog was ≥7 in at least 4/5 individuals from at least one group. These values were used as input for random forests (Breiman, 2001), plotting, WGCNA (Langfelder and Horvath, 2008) and clustering algorithms. DESeq2 (Love et al., 2014) was then used to identify differentially expressed genes (DE genes), defined as those with likelihood ratio test (LRT) adjusted *p*-value of ≤0.001 across all states. Models for both tests included a term to control for the effect of sex. DESeq2 was also used to calculate “shrunken” log2 fold-changes (Stephens, 2017) for differentially expressed genes and normalized, transformed (rlog) count matrices (Supplementary Tables 2–4).

### Exploratory Cluster Analyses

After processing via ComBat (Johnson et al., 2006) to remove effects of sex, expression data from each tissue were passed to WGCNA (Langfelder and Horvath, 2008) v1.68 for module detection. Parameters were optimized for constructing a signed network (TOMType = “signed,” networkType = “signed”), high sensitivity (deepSplit = 3), and more aggressive than default merging and reassignment (mergeCutHeight = 0.25, reassignThreshold = 1). Additional settings were: minModuleSize = 30, minCoreKME = 0.5, minKMEtoStay = 0.4. Genes from each module were inspected in comparison to their summary profile eigengene. Then, module–trait associations quantification was performed to identify modules that were significantly associated with the measured traits. Modules and module-trait correlation values were color coded in plotting.

### Reference Pattern Clustering

Gene expression patterns were assigned to their final reference clusters by calculating Pearson correlation coefficients. For each brain region, mean expression values were calculated for each gene in each state, and these average expression values were tested for correlation with the ten most common patterns observed in WGCNA modules. Correlated genes (*r* ≥ 0.8) were assigned to the cluster with the highest correlation coefficient; no ties were observed. The remaining genes (*r* < 0.8 to any reference cluster) were grouped into the ‘Unassigned’ cluster. Cluster gene overlaps among brain regions were visualized using VennDiagram (Chen and Boutros, 2011) in R.

### Alternative Splicing Analysis

All reads that were at least 20 nt after trimming (described above) were aligned to the HiC genome using STAR (Dobin et al., 2012) in two-pass mode with settings: –limitSjdbInsertNsj 2000000, –outSAMattributes NH HI AS nM MD, –alignSJoverhangMin 8 on the second pass. The STAR alignments were then analyzed using MAJIQ Builder to assemble splice graphs and MAJIQ Quantifier to identify changes in relative local splice variation (LSV) abundance (delta PSI = dPSI) between states (Vaquero-Garcia et al., 2016). LSVs with significant changes, defined as a 99.9% probability of a dPSI of at least 0.2 in any pairwise state comparison, were visualized by separately plotting the abundance of the SA-dominant splice junction and retained intron (if present) relative to SA using ComplexHeatmap (Gu et al., 2016) in R. Reference pattern clustering was performed as described for gene expression data. Protein domains in alternative splicing isoforms were identified using HMMER version 3.3^2^ on version 32.0 of the PFAM database (El-Gebali et al., 2018). For intron retention splice site analysis, temperature-dependent retained intron events with 90% probability of a dPSI of at least 0.2 were identified and the sequences surrounding their 5′ and 3′ splice sites were passed to splice site strength prediction algorithms MaxEntScan (Yeo and Burge, 2004) and HBond (Erkelenz et al., 2018). All non-overlapping non-differentially retained introns described by MAJIQ were used as the control.

### Data Exploration

In addition to data availability in the Supplementary Datasheets 2–4 and Supplementary Tables 2–4, the RNA sequencing analyses as described above are integrated with the new genome assembly, and transcriptome annotation into an interactive R Shiny browser, squirrelBox, which is hosted online^3^ or can be run locally^4^ ^,5^. squirrelBox enables data filtering, query, high quality plotting, and basic GO-term and *k*-mer enrichment analyses. Gene set distribution along the newly improved genome is powered by genomic interval manipulation via valr (Riemondy et al., 2017) and JavaScript-interfacing via Biocircos (Cui et al., 2016). squirrelBox is intentionally designed to be flexible to input datasets and is easily adaptable to other data exploration needs.

### Sequence/Motif Analysis and Gene Ontology Enrichment

Additional analyses were carried out in R, heavily utilizing Bioconductor packages. The newly built genome assembly was forged into a BSgenome data package, which enables quick queries of coding and untranslated sequences. In addition, *k*-mer counting and enrichment statistical analysis are achieved through the R package transite (Krismer et al., 2020). Annotations of *k*-mer and motifs for potentially linked RNA-binding proteins or miRNAs were collected from multiple publicly available annotation databases. For AU-rich element analysis, AREscores (Spasic et al., 2012) were calculated for 3′UTR sequences of at least 200 nt in length via the web interface^6^. Initial GO-term enrichments were found using DAVID for comparison of individual cluster gene lists to the human gene background (Supplementary Table 6, Huang et al., 2009); these were used to annotate the cluster heatmaps. GO-term enrichment analysis in squirrelBox is conducted via Fisher exact test on GO-term collections from the Molecular Signatures Database with three background choices: all human, all annotated ground squirrel, and pass-filter brain-detected genes.

### Data Re-calculations for Comparison to Previous Work

Gene names and log2FC_ashr values for all pairwise DE genes with wald_padj < 0.05 were selected and log2_FC values converted to values for IBA_vs_SpD, SA_vs_SpD, IBA_vs_SA and IBA_vs_LT. Mean values for gene counts for APR, OCT, TOR, and IBA and their pairwise *p*-values were merged [Supplementary Tables 1, 2 in Schwartz et al. (2013)], and then selected for the appropriate Benjamini-adjusted pvalue according to the values in Supplementary Table 3 to get padj < 0.05. The mean fold change was calculated for pairs IBA/APR, OCT/APR, IBA/OCT and IBA/TOR. Correlation and *p*-values were determined in R using cor.test for the DE hypothalamus genes recovered in both studies, as well as for common genes DE in forebrain (this study) and cortex (Schwartz et al., 2013).

## Data Availability Statement

The datasets presented in this study can be found in online repositories. The names of the repository/repositories and accession number(s) can be found in the article/ Supplementary Material.

## Ethics Statement

Ethical review and approval was not required for the animal study because all of the materials used for this work came from a frozen tissue bank.

## Author Contributions

RF and AG analyzed data and drafted and reviewed manuscript. KG prepared DNA, analyzed data, and drafted and reviewed manuscript. KR analyzed data and reviewed manuscript. LE collected tissues and reviewed manuscript. JH supervised data analysis and reviewed manuscript. CB reviewed manuscript. SM designed study, prepared RNA, analyzed data, and drafted and reviewed manuscript. All authors contributed to the article and approved the submitted version.

## Conflict of Interest

KG is CSO for Fauna Bio and SM and CB serve on its SAB. The remaining authors declare that the research was conducted in the absence of any commercial or financial relationships that could be construed as a potential conflict of interest.

## Footnotes

1 http://snap.cs.berkeley.edu

2 hmmer.org

3 https://raysinensis.shinyapps.io/squirrelBox/

4 https://github.com/rnabioco/squirrelbox

5 https://hub.docker.com/r/raysinensis/squirrelbox

6 http://arescore.dkfz.de/arescore.pl
