# Supplementary material for: Dynamic RNA Regulation in the Brain Underlies Physiological Plasticity in a Hibernating Mammal
Source: Front Physiol. 2021 Jan 18;11:624677. doi: 10.3389/fphys.2020.624677 (PMC7848201; doi:10.3389/fphys.2020.624677)
Supplement: Supplementary file 7 [file Data_Sheet_1.pdf]

Supplementary Data Sheet 1.
Supplementary Methods, List of Supplementary Files, and Supplementary Table Legends

Supplementary Methods

Brain dissection. Following exsanguination and perfusion with ice cold saline, the ground squirrel was decapitated with a sharp guillotine at the base of the neck, and multiple tissues were dissected on metal plates placed directly on ice for continuous cooling. Tissues were frozen by submerging each in liquid nitrogen immediately upon dissection and then stored continuously at -70°C until use. Here we describe just the dissection of the three brain regions used in the present study which were all done by LEE to maximize reproducibility. For reference, the thyroid gland remained in the head portion after decapitation. The skin on top of the skull was bisected and peeled away laterally to expose the skull. Bone cutting, blunt-tipped rongeurs were used to remove the skull, beginning at the caudal and dorsal-most point on the skull, cutting directly adjacent to and parallel to the spinal cord forward along the top of the skull to the nose. The top of the skull was further opened using the rongeurs, surgical scissors, and forceps. The brain was carefully scooped out with a blunt spatula, detaching the pituitary gland from the hypothalamus and leaving it to be retrieved from the skull base. The ground squirrel hypothalamus is similar to the rat hypothalamus in appearance and position, forming a flat but distinct pad in the middle of the ventral surface of the brain. An ~65mg ovoid piece of brain tissue surrounding the torn-away infundibulum was dissected with a scalpel as the hypothalamus.

The midbrain portion of the brainstem was separated from the rest of the brain by blunt dissection. The cerebellum was removed, and the resulting brainstem was further dissected into three sections that were flash frozen and stored separately. From rostral to caudal, these were the midbrain, the pons, and the medulla. The cessation of narrowing as the medulla merges with the spinal cord was used as the caudal boundary of the medulla, resulting in an ~85mg, y-shaped tissue segment.

The remaining brain, that is the entire telencephalon and all of the diencephalon minus the hypothalamus, was bisected using a single-edged razor blade through the longitudinal fissure. The two hemispheres were frozen separately. For RNA preparation, the right hemisphere was pulverized in liquid nitrogen using a mortar and pestle. The resulting tissue powder was used for both protein (Hindle and Martin, 2013) and RNA preparation (Riemondy et al., 2018).

List of Files Submitted as Supplementary Material

- **Supplementary Data Sheet 1**. A (this) PDF file containing detailed information regarding Supplementary Materials. It includes brain dissection details, a list of Supplementary Files and legends for Supplementary Tables.
- **Supplementary Data Sheet 2**. A PDF file containing all of the Supplementary Figures, 1-22.

- **Supplementary Data Sheet 3**. A csv file containing full DESeq2 analysis of forebrain RNA-seq data (individual samples are denoted by the animal number preceded by "B").
- **Supplementary Data Sheet 4**. A csv file containing full DESeq2 analysis of hypothalamus RNA-seq data (individual samples are denoted by the animal number preceded by "H").
- **Supplementary Data Sheet 5**. A csv file containing full DESeq2 analysis of medulla RNA-seq data (individual samples are denoted by the animal number preceded by "M").
- **Supplementary Table 1**. Excel file with two tabs summarizing metadata for 1) the sample, sequencing read and alignment characteristics of 90 RNA-seq libraries; and 2) animal and housing parameters which relate to Supplementary Figures 2-4.
- **Supplementary Table 2**. Excel file of DE genes in hibernating 13-lined ground squirrel forebrain. Eight tabs report DESeq2 analysis of RNA-seq data including pseudocounts, statistical test results and fold change for sequential pairs. Relates to Figure 4A and Supplementary Figure 2.
- **Supplementary Table 3**. Excel file of DE genes in hibernating 13-lined ground squirrel hypothalamus. Eight tabs report DESeq2 analysis of RNA-seq data including pseudocounts, statistical test results and fold change for sequential pairs. Relates to Figure 4B and Supplementary Figure 2.
- **Supplementary Table 4**. Excel file of DE genes in hibernating 13-lined ground squirrel medulla. Eight tabs report DESeq2 analysis of RNA-seq data including pseudocounts, statistical test results and fold change for sequential pairs. Relates to Figure 4C and Supplementary Figure 2.
- **Supplementary Table 5**. Excel file summarizing DAVID gene enrichment analysis by cluster for each brain region (three tabs). Relates to Supplementary Figures 7-9.
- **Supplementary Table 6**. Excel file reports cell-specific marker genes in mouse hypothalamus that were detected and DE in ground squirrel hypothalamus.
- **Supplementary Table Legends**

Supplementary Table Legends

Supplementary Table 1. Data summary. Excel file with two tabs.
RNAseqlibrary_data tab, columns report the: A) off-sequencer fastq_id; B) sample_title, which is the sample name in the GEO record (GSE106947); C) sample_id (simplest, one letter, B, H or M, to distinguish brain region, forebrain, hypothalamus and medulla, respectively, plus the animal number) ; D) brain region; E) seqread_length, in nucleotides; F) off_sequencer_fastq, number of read pairs; G) number of readpairs after_trimming; G) %mtDNA , percent of readpairs aligning to 13-lined ground squirrel mitochondrial DNA (Hampton et al., 2011); I) salmon_input, number of readpairs; J) salmon_pseudoaligned, number of readpairs; K) %pseudoaligned, fraction of original paired-end reads pseudoaligned to HiC_Itri_2.
Animal_data tab, columns report the: A) Group, physiological state as defined in Figure 1; B) Animal, unique sample identifier; C) Sex, M, male, or F, female; D) Heterotherm, logical indicating if the animal was cycling through multiday torpor and arousal; E) FoodAvailable, logical; F) BodyMassAtMoveToHibernaculum, g; G) DateMovedToHibernaculum; H) MassAtEuthanasia, g; I) FractionBodyMassLostInHibernaculum; J) DateEuthanized; K) Housing,

whether animal was in standard laboratory animal housing or in the hibernaculum at the time of euthanasia; L) HrsLight, number of hours/day with lights on; M) AmbientTemperature in °C; N) DaysInHibernaculum; O) BodyTemperature in °C; P) TimeofDay. The effect of these parameters on differential gene expression was tested using WGCNA, see Supplementary Figures 3, 4 and 5 in Supplementary Data Sheet 2. These RNA-seq libraries were examined previously for A-to-I editing (Riemondy et al., 2018), which demonstrated that genetic relatedness did not define these animal groups. All animals except 55, 65, 68, 153, and 163 were adults (Grabek et al., 2019).

Supplementary Table 2. **Forebrain DE genes**. Excel file with eight tabs.
forebrain_DEgenes, for all genes LRT_padj < 0.001 columns report**:** A-F) information about the gene ID (see Methods); G-AJ) DESeq2 pseudocounts (normalized, transformed (rlog) counts) for each sample; AK-AL) DESeq2 likelihood ratio test result (LRT_pvalue) and adjusted pvalue (LRT_padj); AM) cluster assignment. Tabs **SA_vs_SpD to IBA_vs_SpD**, contain DE genes for each pairwise comparison in Figure 4A. Columns report the unique_gene_symbol (GeneID), log2 fold-change (group1_vs_group2_log2FC_ashr) for that pairwise comparison with its pvalue (group1_vs_group2_wald_pvalue) and adjusted pvalue (group1_vs_group2_wald_padj). See Methods for detailed explanations.

Supplementary Table 3. **Hypothalamus DE genes**. Excel file with eight tabs.
hypothalamus_DEgenes, for all genes LRT_padj < 0.001 columns report**:** A-F) information about the gene ID (see Methods); G-AJ) DESeq2 pseudocounts (normalized, transformed (rlog) counts) for each sample; AK-AL) DESeq2 likelihood ratio test result (LRT_pvalue) and adjusted pvalue (LRT_padj); AM) cluster assignment. Tabs **SA_vs_SpD to IBA_vs_SpD**, contain DE genes for each pairwise comparison in Figure 4B. Columns report the unique_gene_symbol (GeneID), log2 fold-change (group1_vs_group2_log2FC_ashr) for that pairwise comparison with its pvalue (group1_vs_group2_wald_pvalue) and adjusted pvalue (group1_vs_group2_wald_padj). See Methods for detailed explanations.

Supplementary Table 4. **Medulla DE genes**. Excel file with eight tabs.
medulla_DEgenes, for all genes LRT_padj < 0.001 columns report**:** A-F) information about the gene ID (see Methods); G-AJ) DESeq2 pseudocounts (normalized, transformed (rlog) counts) for each sample; AK-AL) DESeq2 likelihood ratio test result (LRT_pvalue) and adjusted pvalue (LRT_padj); AM) cluster assignment. Tabs **SA_vs_SpD to IBA_vs_SpD**, contain DE genes for each pairwise comparison in Figure 4C. Columns report the unique_gene_symbol (GeneID), log2 fold-change (group1_vs_group2_log2FC_ashr) for that pairwise comparison with its pvalue (group1_vs_group2_wald_pvalue) and adjusted pvalue (group1_vs_group2_wald_padj). See Methods for detailed explanations.

Supplementary Table 5. Gene enrichment analysis. Excel file with three tabs, one for each brain region containing David (Huang et al., 2009a;Huang et al., 2009b) output for gene ontology (GO) terms associated with each cluster (see also Supplementary Figures 7-9).

Supplementary Table 6. Ground squirrel hypothalamus genes associated with multiple cell types in mouse hypothalamus. Columns A, C, D and E were taken directly from Table S1 in (Chen et al., 2017); column B reports which of the mouse marker genes in column C that were associated with hypothalamus cell types in column A were detected and DE (if bold) in our ground squirrel hypothalamus RNA-seq libraries.

References Cited

Chen, R., Wu, X., Jiang, L., and Zhang, Y. (2017). Single-Cell RNA-Seq Reveals Hypothalamic Cell Diversity. *Cell Rep* 18**,** 3227-3241.

Grabek, K.R., Cooke, T.F., Epperson, L.E., Spees, K.K., Cabral, G.F., Sutton, S.C., Merriman, D.K., Martin, S.L., and Bustamante, C.D. (2019). Genetic variation drives seasonal onset of hibernation in the 13-lined ground squirrel. *Communications biology* 2**,** 478-478.

Hampton, M., Melvin, R.G., Kendall, A.H., Kirkpatrick, B.R., Peterson, N., and Andrews, M.T. (2011). Deep sequencing the transcriptome reveals seasonal adaptive mechanisms in a hibernating mammal. *PLoS ONE* 6**,** e27021.

Hindle, A.G., and Martin, S.L. (2013). Cytoskeletal regulation dominates temperature-sensitive proteomic changes of hibernation in forebrain of 13-lined ground squirrels. *PLoS ONE* 8**,** e71627.

Huang, D.W., Sherman, B.T., and Lempicki, R.A. (2009a). Systematic and integrative analysis of large gene lists using DAVID bioinformatics resources. *Nat Protoc* 4**,** 44-57.

Huang, D.W., Sherman, B.T., Zheng, X., Yang, J., Imamichi, T., Stephens, R., and Lempicki, R.A. (2009b). Extracting biological meaning from large gene lists with DAVID. *Curr Protoc Bioinformatics* Chapter 13**,** Unit 13 11.

Riemondy, K.A., Gillen, A.E., White, E.A., Bogren, L.K., Hesselberth, J.R., and Martin, S.L. (2018). Dynamic temperature-sensitive A-to-I RNA editing in the brain of a heterothermic mammal during hibernation. *RNA* 24**,** 1481-1495.
