# Supplementary material for: Dynamic RNA Regulation in the Brain Underlies Physiological Plasticity in a Hibernating Mammal
Source: Front Physiol. 2021 Jan 18;11:624677. doi: 10.3389/fphys.2020.624677 (PMC7848201; doi:10.3389/fphys.2020.624677)
Supplement: Supplementary file 8 [file Data_Sheet_2.pdf]

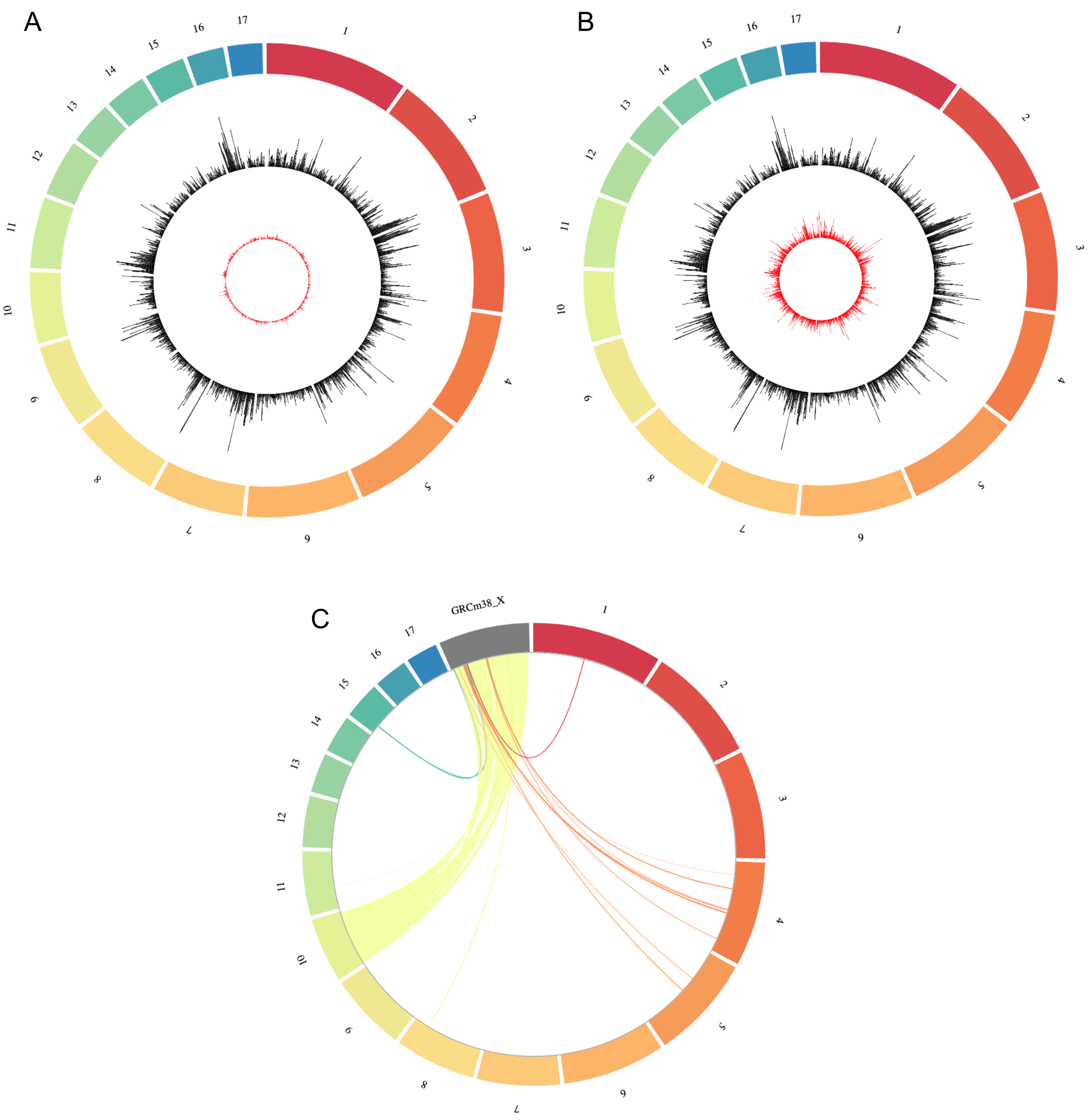

Supplementary Figure 1. Circos plots of the 17 longest contigs from the improved 13-lined ground squirrel genome, HiC_Itri_2. **A, B**) Gene distributions compare previously reported genes (n=28525, plotted in black) and novel genes in red for: **A**) genes with at least 2 exons and orf length ≥ 100aa (2809 genes); or **B**) all genes (n=14356). **C**) Synteny analysis comparing ground squirrel HiC_Itri_2 contigs 1-17 to the mouse X chromosome (GRCm38.X).

Supplementary Figure 2. Pairwise DE genes in three brain regions

**Forebrain**	SAvsSpD	IBAvsSA	IBAvsEnt	EntvsLT	ArvsLT	IBAvsAr	IBAvsSpD
total DE	2	411	790	31	12	1539	414
# increased	2	229	539	26	4	1026	224
# decreased	0	182	251	5	8	513	190
#≥2x increased	0	4	11	4	0	42	2
#≥2x decreased	NA	1	6	1	0	4	4
fraction DE≥2x	NA	0.012	0.022	0.161	NA	0.030	0.014
Max FC increased	0.70	4.29	2.37	3.53	0.87	4.70	3.58
Max FC decreased	NA	-1.01	-2.11	-1.01	-0.63	-1.41	-2.11
GeneID unknown	0	2	13	0	0	46	5
fraction unknown	NA	0.005	0.016	NA	NA	0.030	0.012

**Hypothalmus**	SAvsSpD	IBAvsSA	IBAvsEnt	EntvsLT	ArvsLT	IBAvsAr	IBAvsSpD
total DE	67	1206	1659	52	0	2085	1569
# increased	28	566	1006	40	NA	1323	736
# decreased	39	640	653	12	NA	672	833
#≥2x increased	1	5	1	5	NA	38	1
#≥2x decreased	3	12	2	7	NA	14	9
fraction DE≥2x	0.060	0.014	0.002	0.231	NA	0.025	0.006
Max FC increased	1.76	1.71	1.91	3.40	NA	3.87	2.38
Max FC decreased	-2.47	-2.13	-1.16	-4.02	NA	-5.15	-1.43
GeneID unknown	0	12	31	3	NA	45	16
fraction unknown	NA	0.010	0.019	0.058	NA	0.022	0.010

**Medulla**	SAvsSpD	IBAvsSA	IBAvsEnt	EntvsLT	ArvsLT	IBAvsAr	IBAvsSpD
total DE	5	517	323	28	2	1026	279
# increased	4	182	230	22	1	709	96
# decreased	1	335	93	6	1	317	183
#≥2x increased	2	0	3	8	0	53	0
#≥2x decreased	0	10	1	1	0	14	5
fraction DE≥2x	0.400	0.019	0.012	0.321	NA	0.065	0.018
Max FC increased	1.22	0.84	1.38	3.29	0.64	3.39	0.82
Max FC decreased	-0.47	-1.46	-1.39	-3.48	-0.77	-4.56	-1.22
GeneID unknown	0	7	7	4	0	38	9
fraction unknown	NA	0.014	0.022	0.143	NA	0.037	0.032

Numbers report number of genes with reference to first of the two states (Figures 1, 4A-C) in each pair compared. DE, differentially expressed; q<0.001; Max FC, maximum fold change; GeneID unknown, no homolog or likely homolog to an identified human gene, although, as indicated in the text, the homologous genomic region may be apparent; NA, not applicable. Relates to Supplementary Material Tables 2-4 and Data Sheets 2-4.

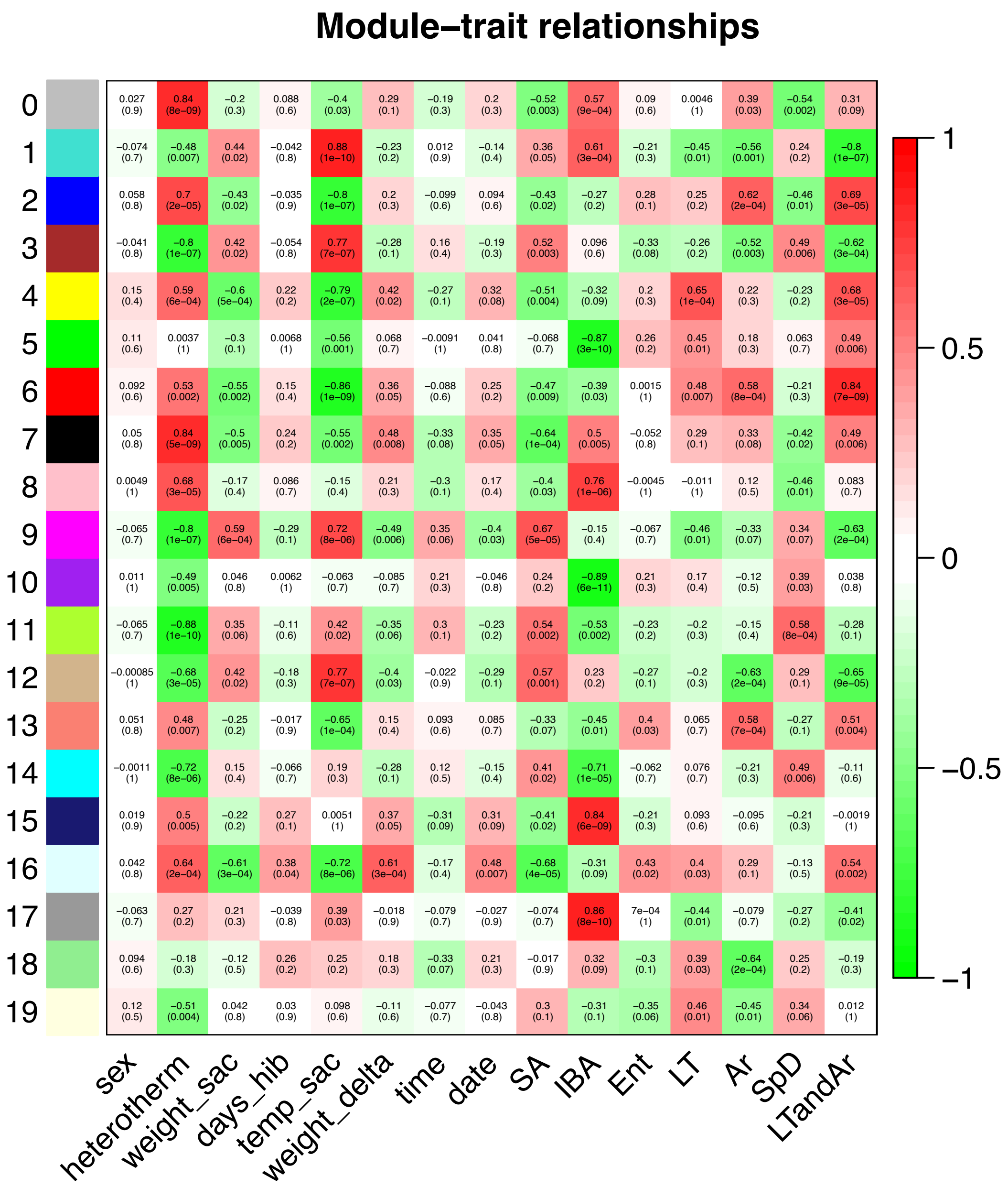

Supplementary Figure 3. WGCNA cluster-phenotype correlations in forebrain. Colors (n=19) on the left represent clusters of co-expressed genes. Numbers in each box report the correlation (top number) and its significance (bottom number, in parenthesis) between each cluster and the phenotypes listed across the bottom. These relate to the information in Supplementary Material Table 1 on the Animal_data tab where weight_sac is body mass at time of tissue collection (MassAtEuthanasia), days_hib are the number of days in the hibernaculum (DaysInHibernaculum), temp_sac is the body temperature at time of tissue collection (BodyTemperature), weight_delta is change in body mass from the time moved to the hibernaculum until the time of tissue collection (FractionBodyMassLostInHibernaculum), time is time of tissue collection (TimeOfDay), date is the date of tissue collection (DateEuthanized) and physiological states are as defined in Group.

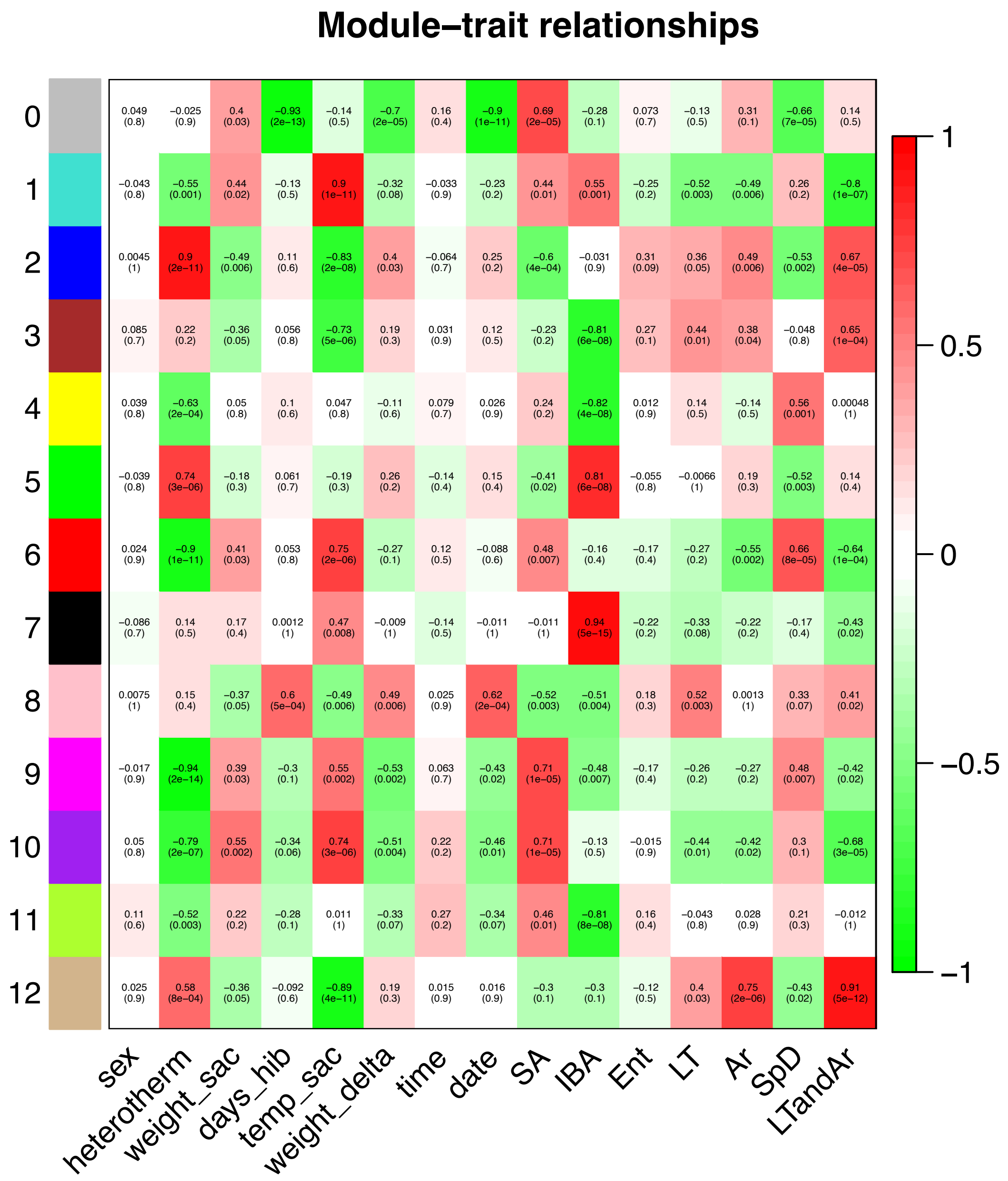

Supplementary Figure 4. WGCNA cluster-phenotype correlations in hypothalamus. Colors (n=12) on the left represent clusters of co-expressed genes. Numbers in each box report the correlation (top number) and its significance (bottom number, in parenthesis) between each cluster and the phenotypes listed across the bottom. See legend to Supplementary Figure 4 for detailed definitions of those phenotypes which are based on the information in Supplementary Material Table 1.

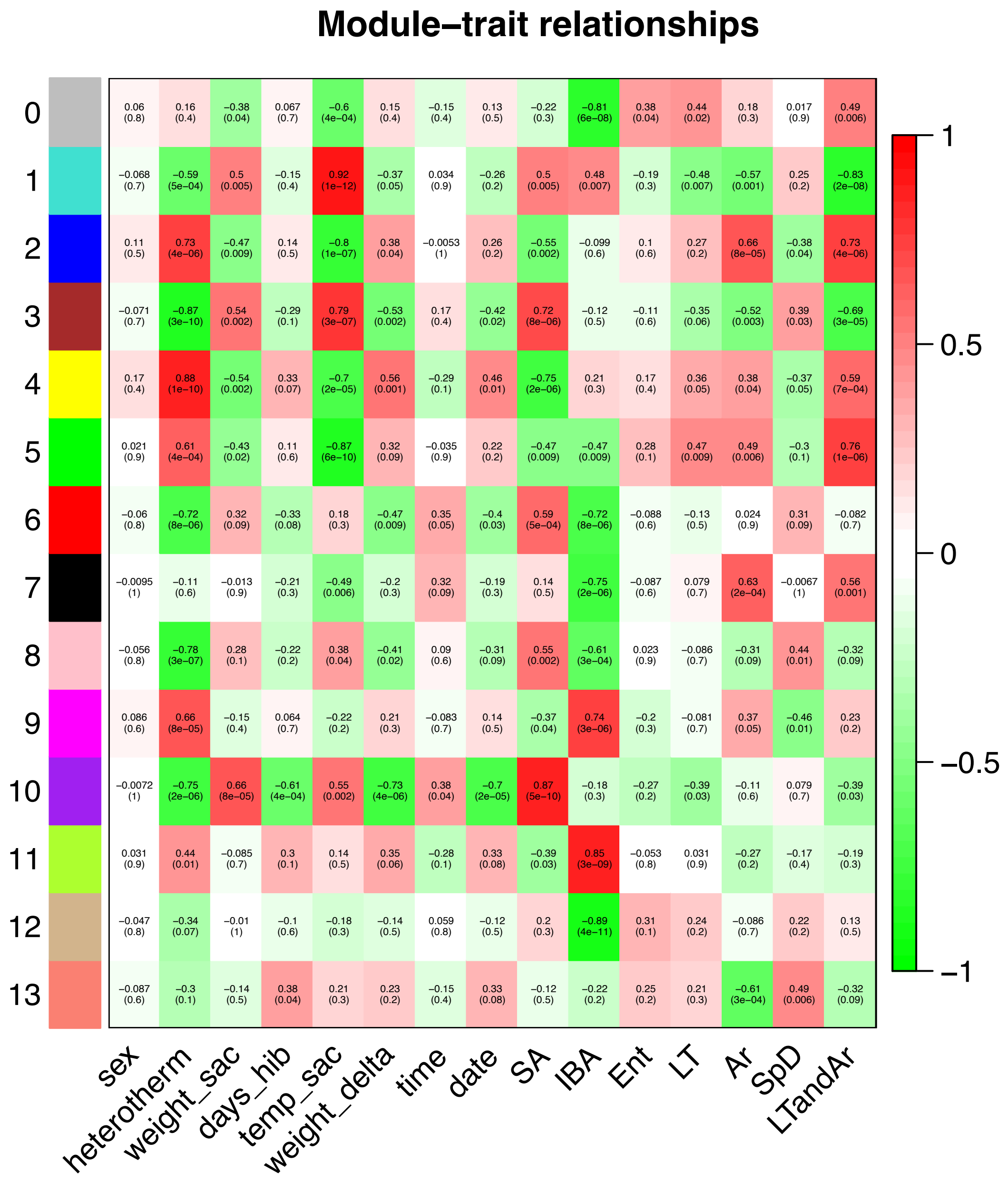

Supplementary Figure 5. WGCNA cluster-phenotype correlations in medulla. Colors (n=13) on the left represent clusters of co-expressed genes. Numbers in each box report the correlation (top number) and its significance (bottom number, in parenthesis) between each cluster and the phenotypes listed across the bottom. See legend to Supplementary Figure 4 for detailed definitions of the phenotypes, which are based on the information in Supplementary Material Table 1.

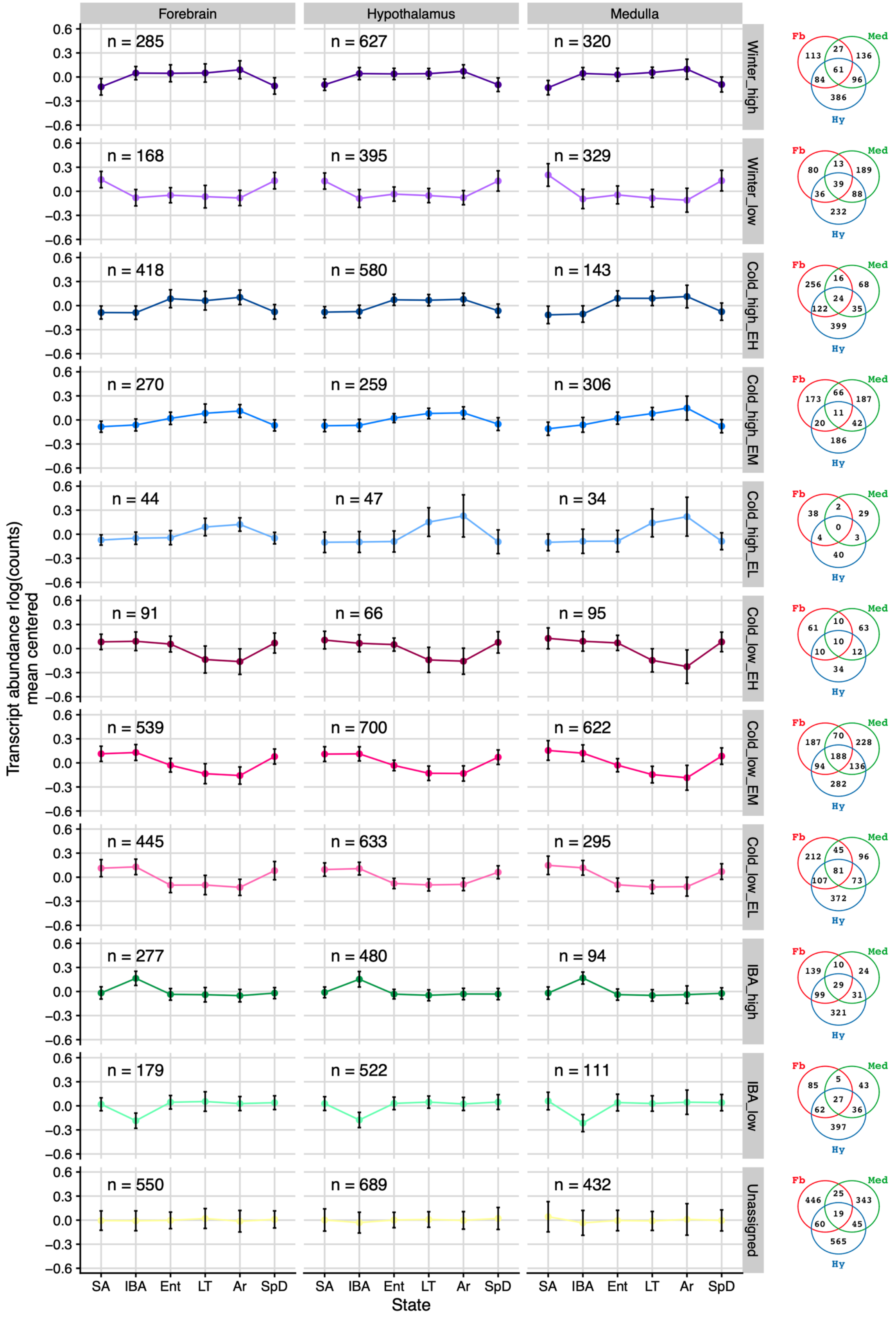

Supplementary Figure 6. Mean expression patterns for all DE genes in three brain regions. Genes were clustered by similarity to the 10 most common relative-abundance patterns (see Methods). Error bars represent standard deviation. The Venn diagram to the right of each cluster reports the number of unique and common genes among Forebrain(Fb), Medulla (Med) and Hypothalamus (Hy).

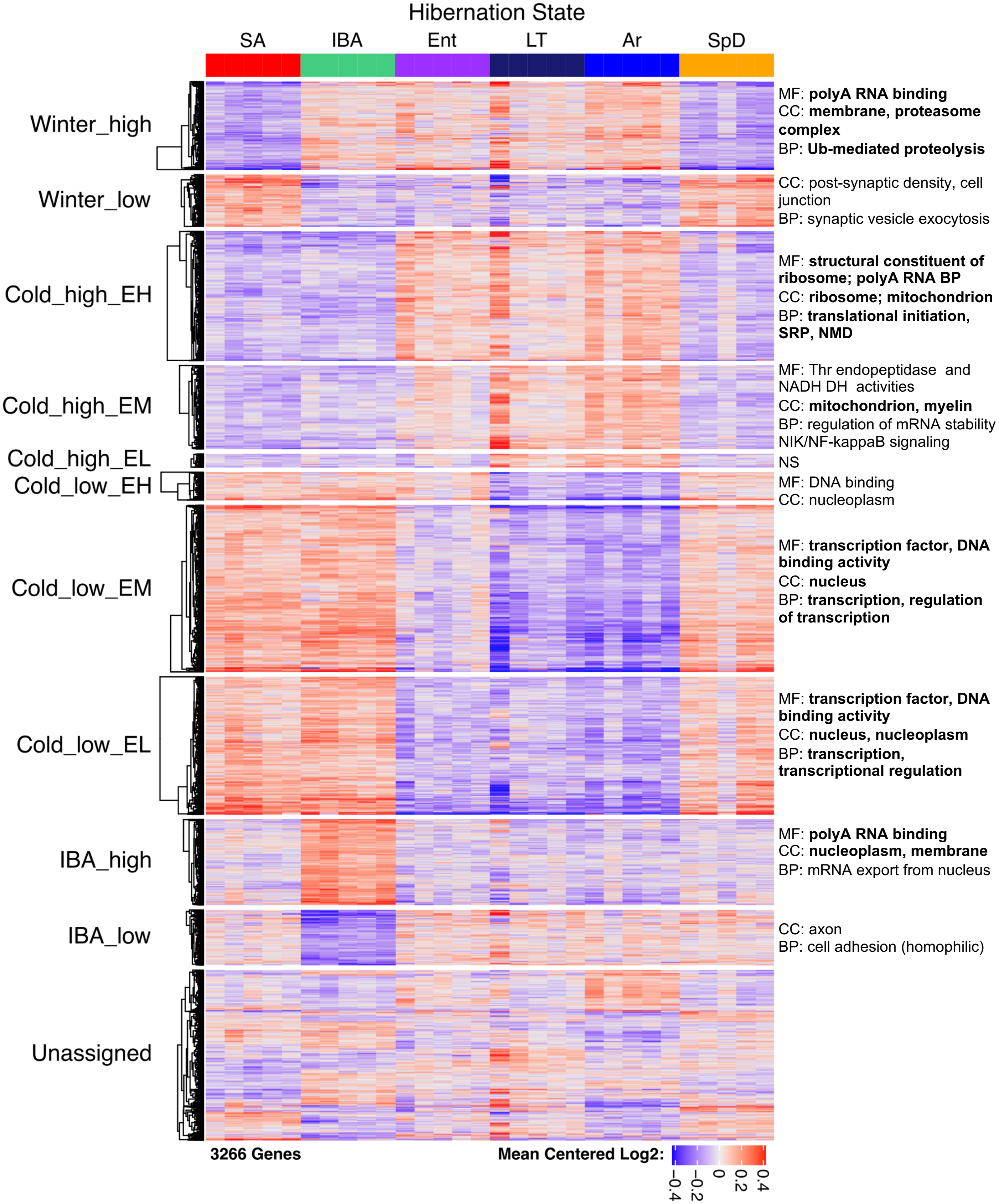

Supplementary Figure 7. Heatmap of clustered forebrain DE genes. Enriched GO terms (MF, molecular function; CC, cellular component; BP, biological process) for each cluster are reported on right ($p \leq 0.001$, bold = $q \leq 0.001$, see also Supplementary Material Table 5). In the molecular function category, protein binding and extracellular exosome are common and omitted here. BP binding protein; DH dehydrogenase; NMD, nonsense-mediated decay; SRP, signal recognition particle; Ub, ubiquitin.

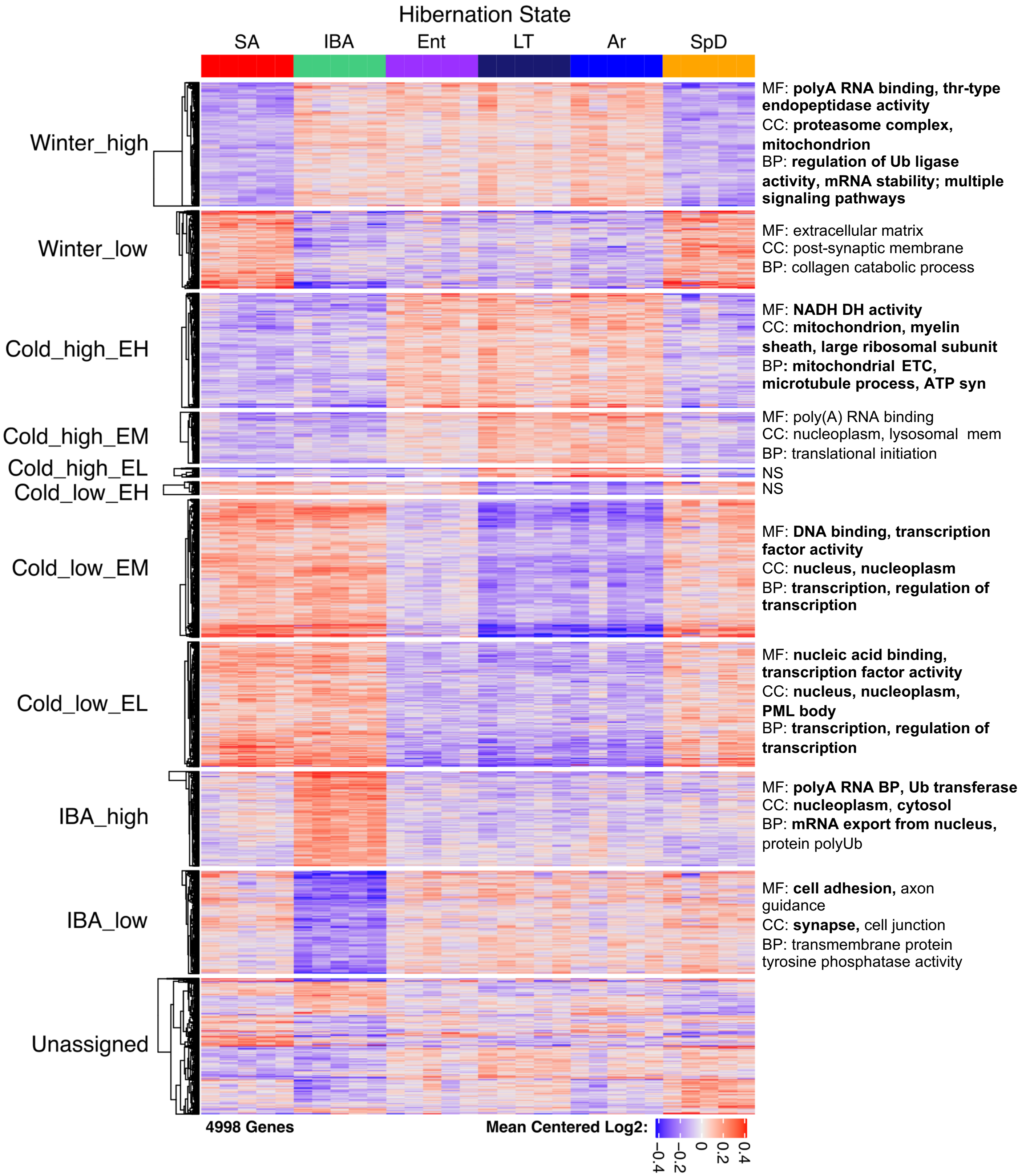

Supplementary Figure 8. Heatmap of clustered hypothalamus DE genes. Enriched GO terms (MF, molecular function; CC, cellular component; BP, biological process) are reported on the right (p ≤ 0.001, bold = q ≤ 0.001, see also Supplementary Material Table 5). In the molecular function category, protein binding and extracellular exosome are common and omitted here. BP binding protein; DH dehydrogenase; ETC, electron transport chain; PML, promyelocytic leukemia; syn, synthase; Ub, ubiquitin.

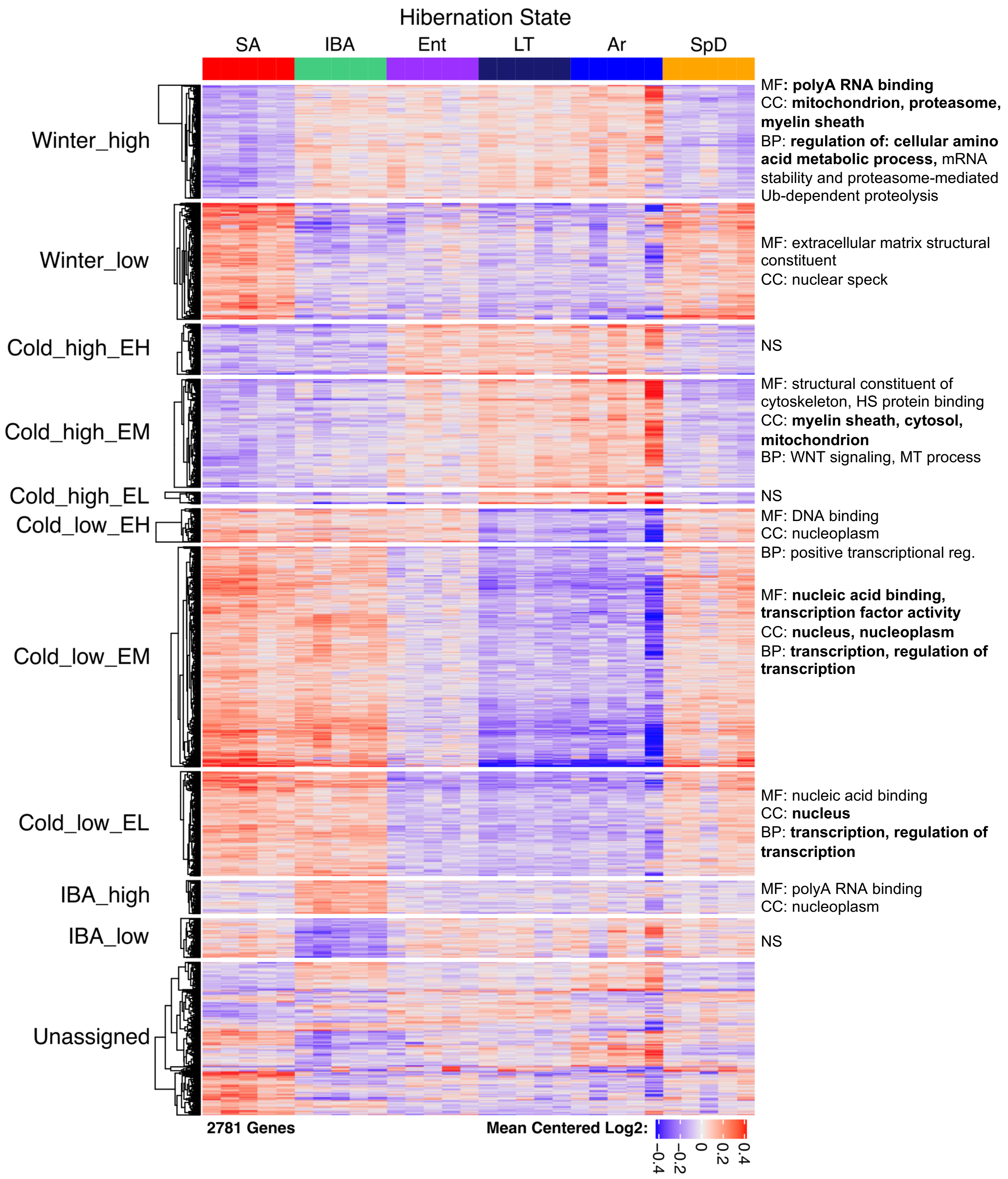

Supplementary Figure 9. Heatmap of clustered medulla genes. Enriched GO terms (MF, molecular function; CC, cellular component; BP, biological process) for each cluster are reported on the right ($p \leq 0.001$, bold = $q \leq 0.001$, see also Supplementary Material Table 5). In the molecular function category, protein binding and extracellular exosome are common and omitted here. MT, microtubule.

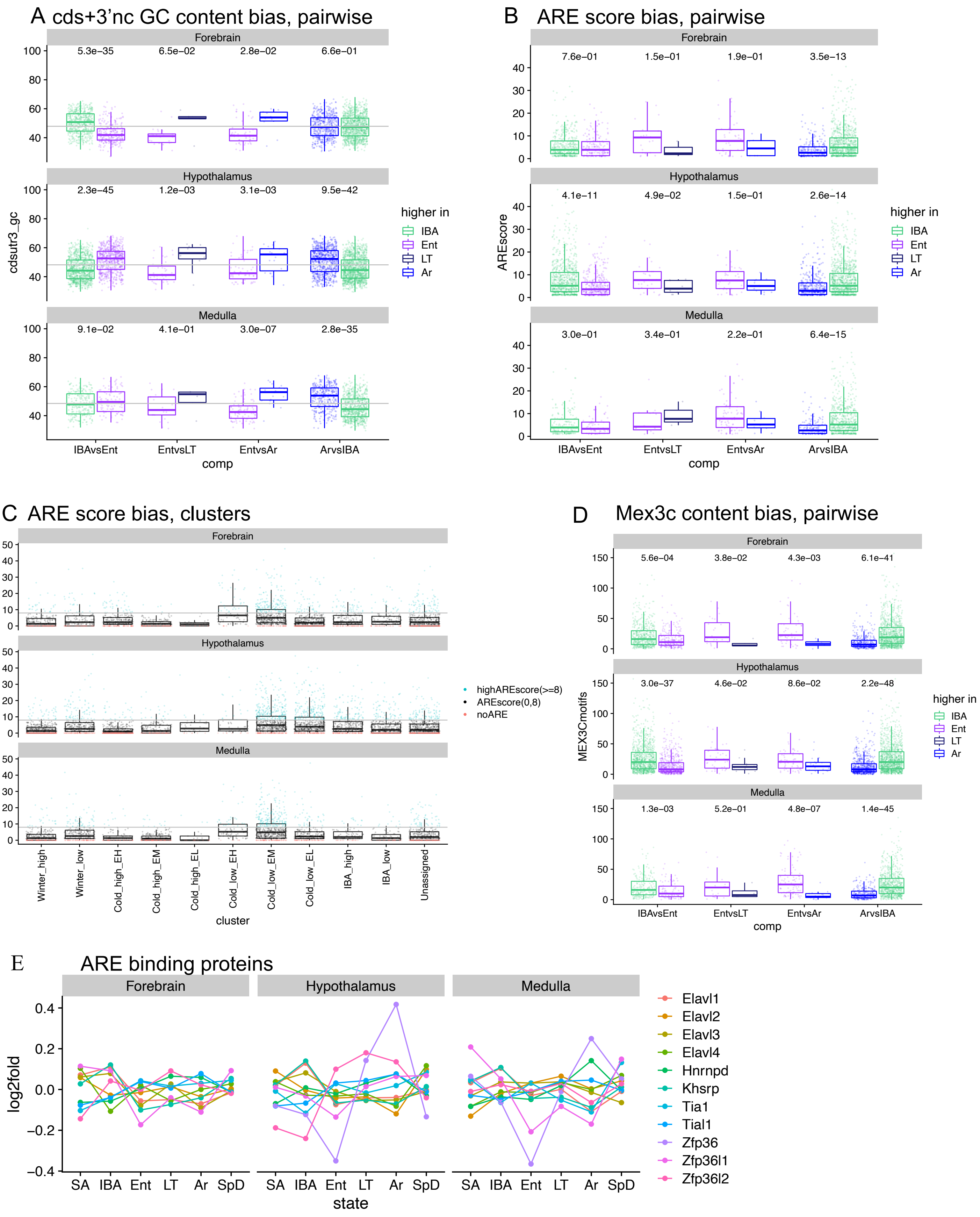
A cds+3'nc GC content bias, pairwise
Forebrain
5.3e−35
6.5e−02
2.8e−02
6.6e−01
Hypothalamus
2.3e−45
1.2e−03
3.1e−03
9.5e−42
Medulla
9.1e−02
4.1e−01
3.0e−07
2.8e−35
cdsutr3_gc
IBAvsEnt
EntvsLT
EntvsAr
ArvsIBA
comp
higher in
IBA
Ent
LT
Ar
B ARE score bias, pairwise
Forebrain
7.6e−01
1.5e−01
1.9e−01
3.5e−13
Hypothalamus
4.1e−11
4.9e−02
1.5e−01
2.6e−14
Medulla
3.0e−01
3.4e−01
2.2e−01
6.4e−15
AREscore
IBAvsEnt
EntvsLT
EntvsAr
ArvsIBA
comp
higher in
IBA
Ent
LT
Ar
C ARE score bias, clusters
Forebrain
Hypothalamus
Medulla
AREscore
Winter_high
Winter_low
Cold_high_EH
Cold_high_EM
Cold_high_EL
Cold_low_EH
Cold_low_EM
Cold_low_EL
IBA_high
IBA_low
Unassigned
cluster
highAREscore(>=8)
AREscore(0,8)
noARE
D Mex3c content bias, pairwise
Forebrain
5.6e−04
3.8e−02
4.3e−03
6.1e−41
Hypothalamus
3.0e−37
4.6e−02
8.6e−02
2.2e−48
Medulla
1.3e−03
5.2e−01
4.8e−07
1.4e−45
MEX3Cmotifs
IBAvsEnt
EntvsLT
EntvsAr
ArvsIBA
comp
higher in
IBA
Ent
LT
Ar
E ARE binding proteins
Forebrain
Hypothalamus
Medulla
log2fold
SA
IBA
Ent
LT
Ar
SpD
state
Elavl1
Elavl2
Elavl3
Elavl4
Hnrnpd
Khsrp
Tia1
Tial1
Zfp36
Zfp36l1
Zfp36l2

Supplementary Figure 10. Sequence features affecting transcript abundance in forebrain, hypothalamus and medulla. **A)** GC content of genes DE in torpor-arousal cycle transitions, as partially presented in Figure 7A; dots represent DE genes in the pairwise comparison indicated below, each assigned to the state where it was increased; boxes delineate the interquartile range, horizontal lines mark the group median, and whiskers indicate the boundaries beyond which sample dots lying outside are outliers. Numbers above are the Wilcoxon test p values and horizontal gray lines represent median GC content of all detected genes in that region. **B)** AREscore for torpor-arousal cycle transitions, calculated using the web interface at http://arescore.dkfz.de/arescore.pl, to predict the destabilizing strength of type-II AU-rich elements for all 3'UTR sequences of coding genes with CDS length >200nt and 3'UTR length >200nt; plot and numbers are as described for panel A. **C)** Dots represent AREscores for genes in the indicated co-expression cluster. Horizontal gray line denotes the cutoff score of 8 used for the bias calculations in Figure 7B. **D)** Mex3c motif content as calculated by scanning for (A/G/U)(G/U)AGN0-8(A/G/U)(A/U)(A/U)(A/G/U)N in the 3'UTR of genes, plot and numbers are as described for panel a and as partially presented in Figure 7C. **E**) Differential expression of mRNAs encoding ARE binding proteins (partially presented in Figure 7D).

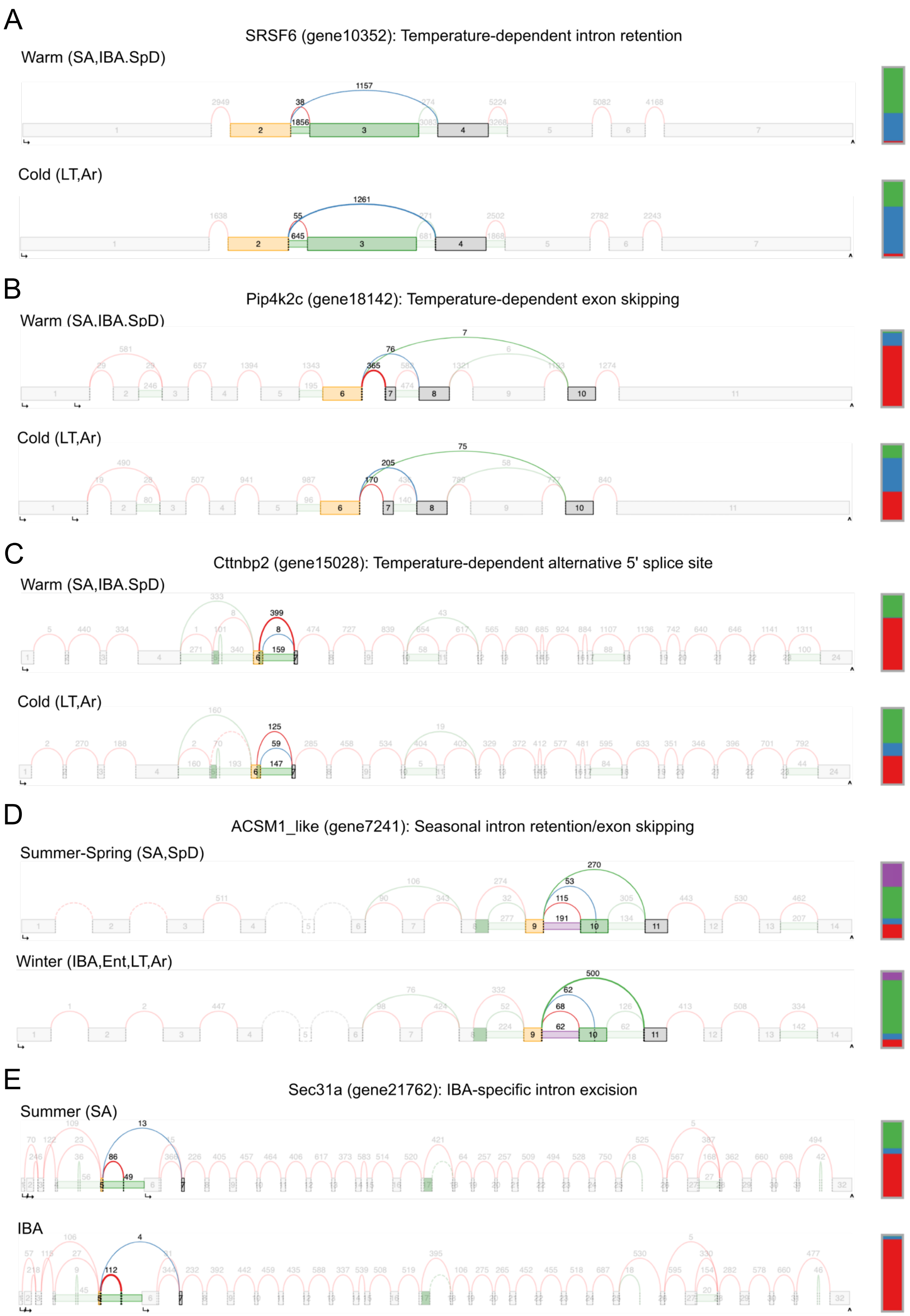

Supplementary Figure 11. Alternative splicing events in hibernation. **A**) *SRSF6*, temperature-dependent intron retention; **B**) *Pip4k2c*, temperature-dependent exon skipping; **C**) *Cttnbp2*, temperature-dependent alternative 5’ splice site; **D**) *ACSM1_like*, seasonal intron retention/exon skipping; and **E**) *Sec31a*, IBA-specific intron retention. Stacked bar graphs on the right show the relative abundance of each color-coded junction in each group. Splicegraphs are from MAJIQ analysis (Vaquero-Garcia et al., 2016) of hypothalamus RNA-seq data, but most of these events are common among all three brain regions.

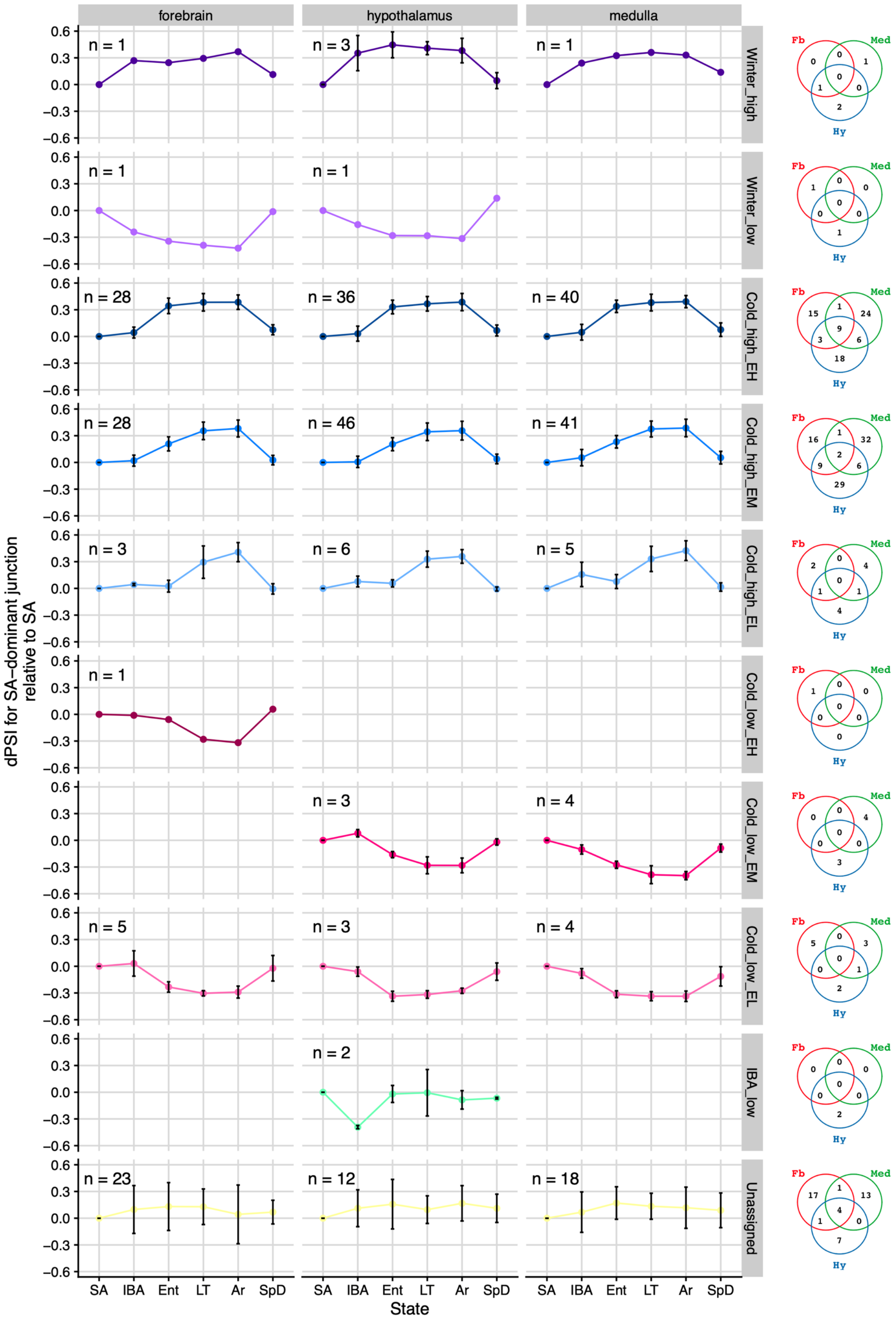

Supplementary Figure 12. Mean alternative splicing patterns for all significant LSVs in three brain regions. dPSI > 0 indicates increased alternative splicing relative to SA (alternative junction becomes more common), while dPSI < 0 indicates decreased alternative splicing relative to to SA. Error bars show standard deviation. Venn diagrams quantify common and unique events among Forebrain (Fb), Medulla (Med) and Hypothalamus (Hy).

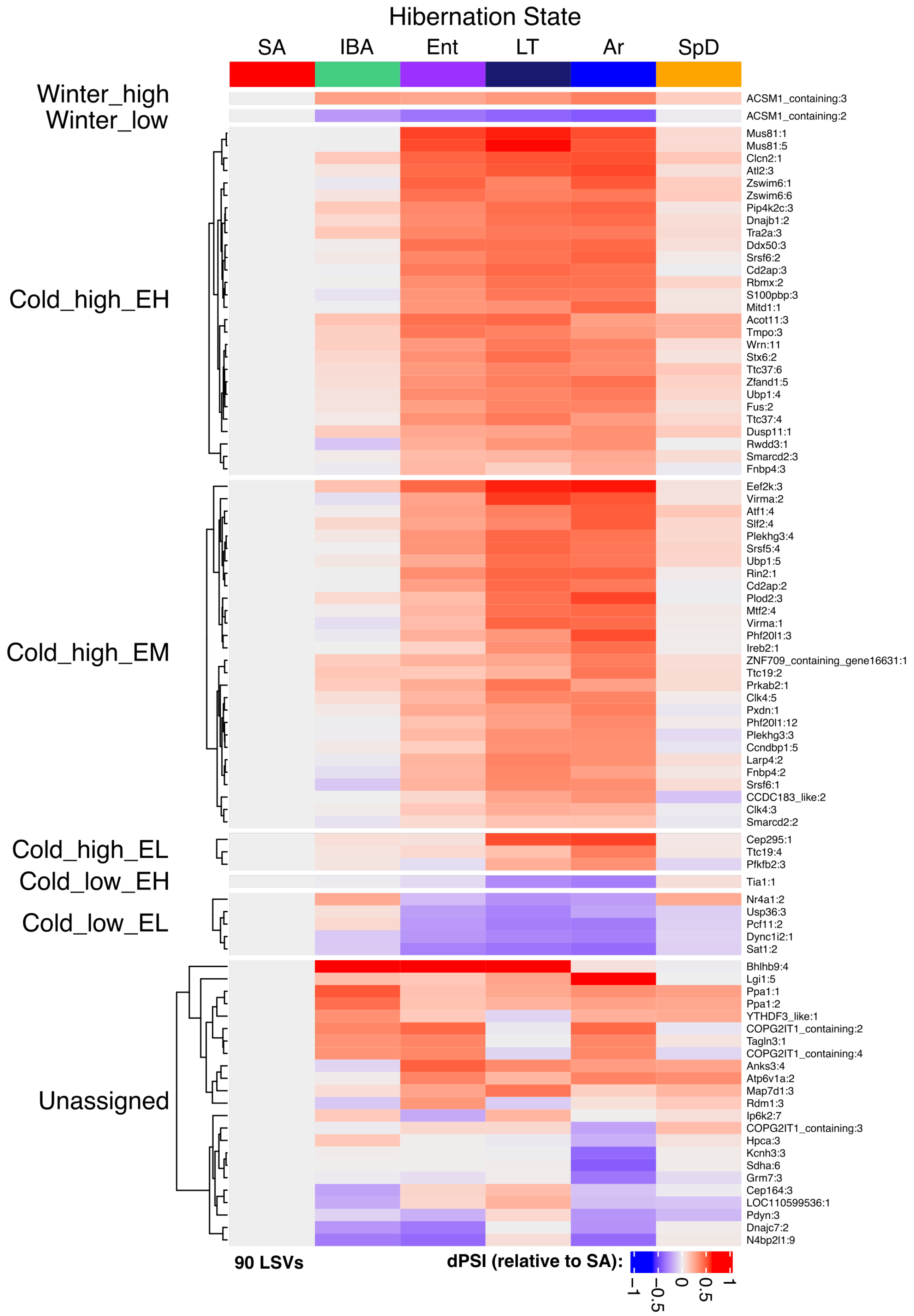

Supplementary Figure 13. Genes with alternative splice sites in forebrain. Heatmap of alternatively spliced LSVs clustered by pattern, each row represents one gene. For each LSV, the abundance of all junctions except the one most commonly observed in SA is plotted relative to SA. dPSI > 0 is increased alternative splicing relative to SA (alternative junction becomes more common), while dPSI < 0 indicates decreased alternative splicing relative to SA. Genes with multiple significant LSVs have numeric suffixes appended to their gene name following a colon.

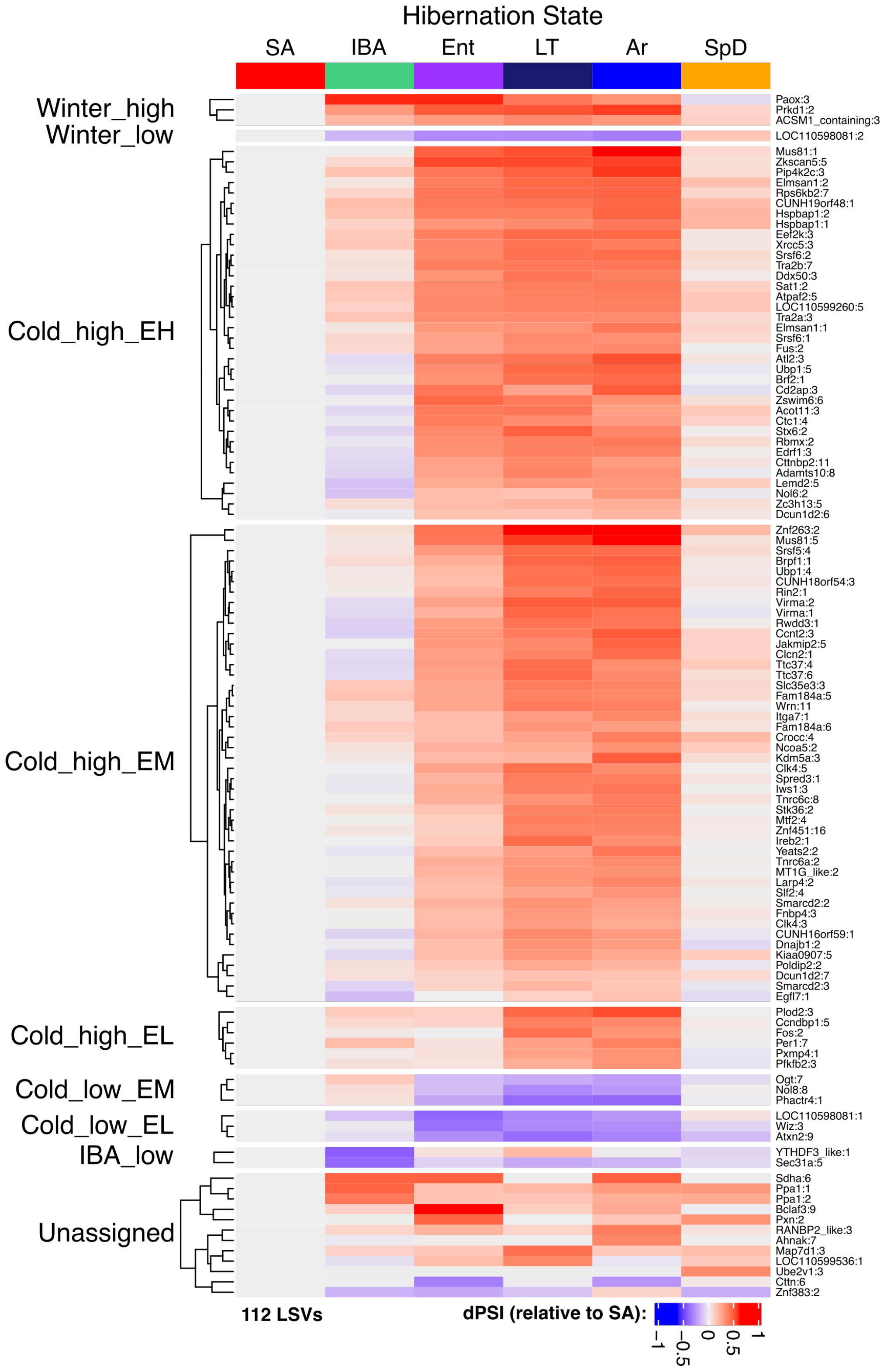

Supplementary Figure 14. Genes with alternative splice sites in hypothalamus. Heatmap of alternatively spliced LSVs clustered by pattern, each row represents one gene. For each LSV, the abundance of all junctions except the one most commonly observed in SA is plotted relative to SA. dPSI > 0 is increased alternative splicing relative to SA (alternative junction becomes more common), while dPSI < 0 indicates decreased alternative splicing relative to SA. Genes with multiple significant LSVs have numeric suffixes appended to their gene name following a colon.

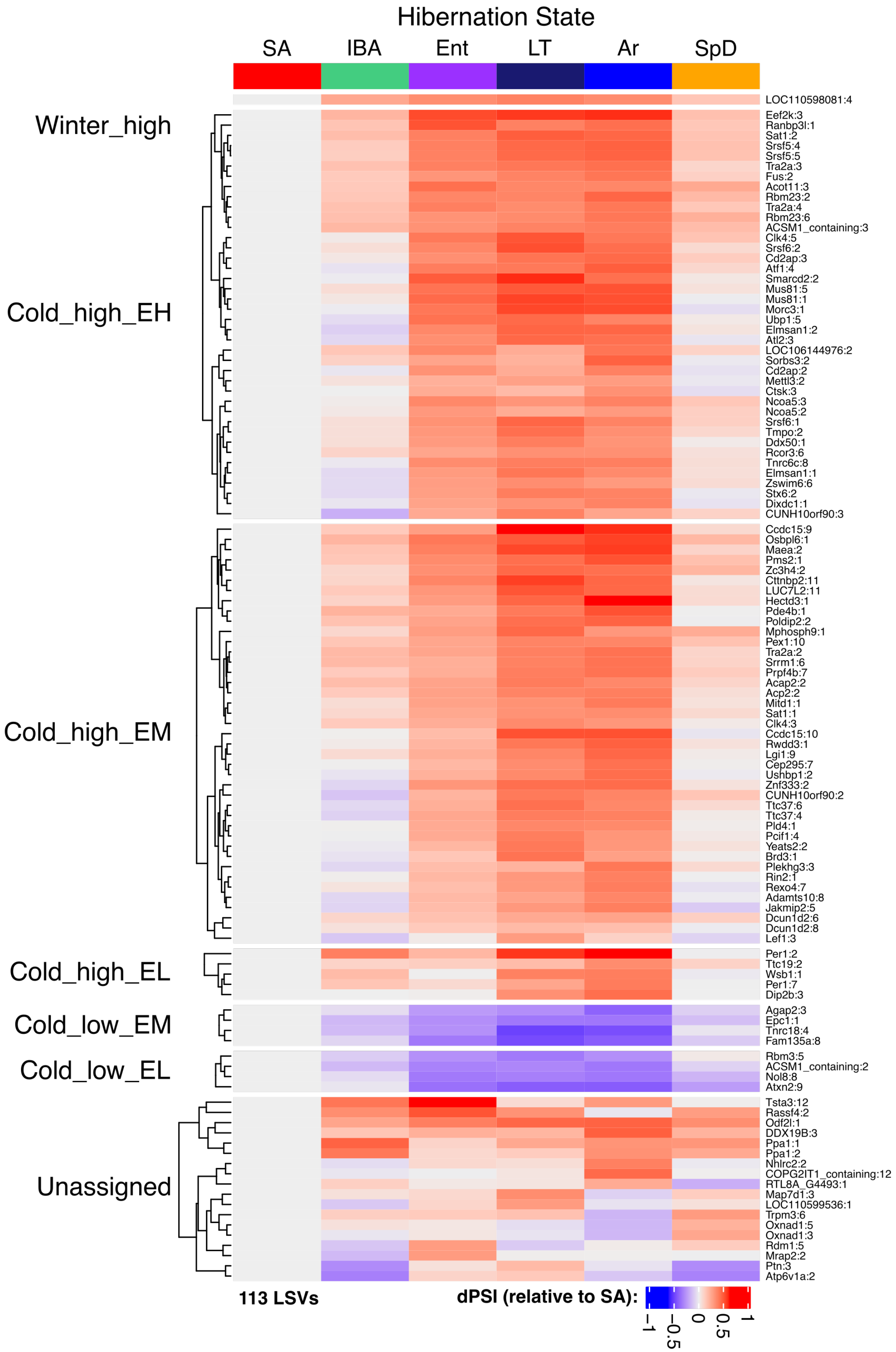

Supplementary Figure 15. Genes with alternative splice sites in medulla. Heatmap of alternatively spliced LSVs clustered by pattern, each row represents one gene. For each LSV, the abundance of all junctions except the one most commonly observed in SA is plotted relative to SA. dPSI > 0 is increased alternative splicing relative to SA (alternative junction becomes more common), while dPSI < 0 indicates decreased alternative splicing relative to SA. Genes with multiple significant LSVs have numeric suffixes appended to their gene name following a colon.

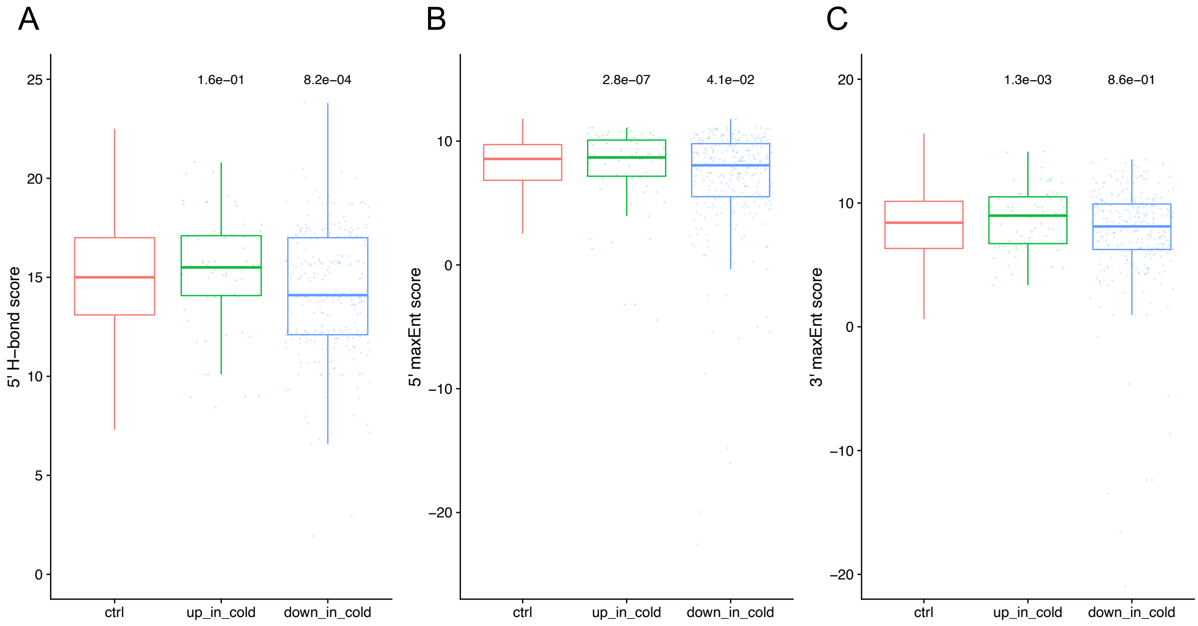

Supplementary Figure 16. Splice site strength of differentially retained introns in warm vs. cold states. Sequences surrounding three groups of introns were analyzed for splice site strength: **A**) H-bond score; **B**) 5'-maximum entropy score; and **C**) 3'-maxiumum entropy score. "ctrl" denotes all non-overlapping, non-differentially retained introns described by MAJIQ; "up_in_cold" denotes increased intron retention in the cold states vs. warm in at least one brain region, "down_in_cold" denotes introns less likely to be retained in the cold. Higher scores for both maxEnt (for 5' splice sites and 3' splice sites) and HBond (for 5' splice sites only) imply stronger and more canonical splice sites. Numbers indicate the p values from Wilcoxon test of control vs. labelled group.

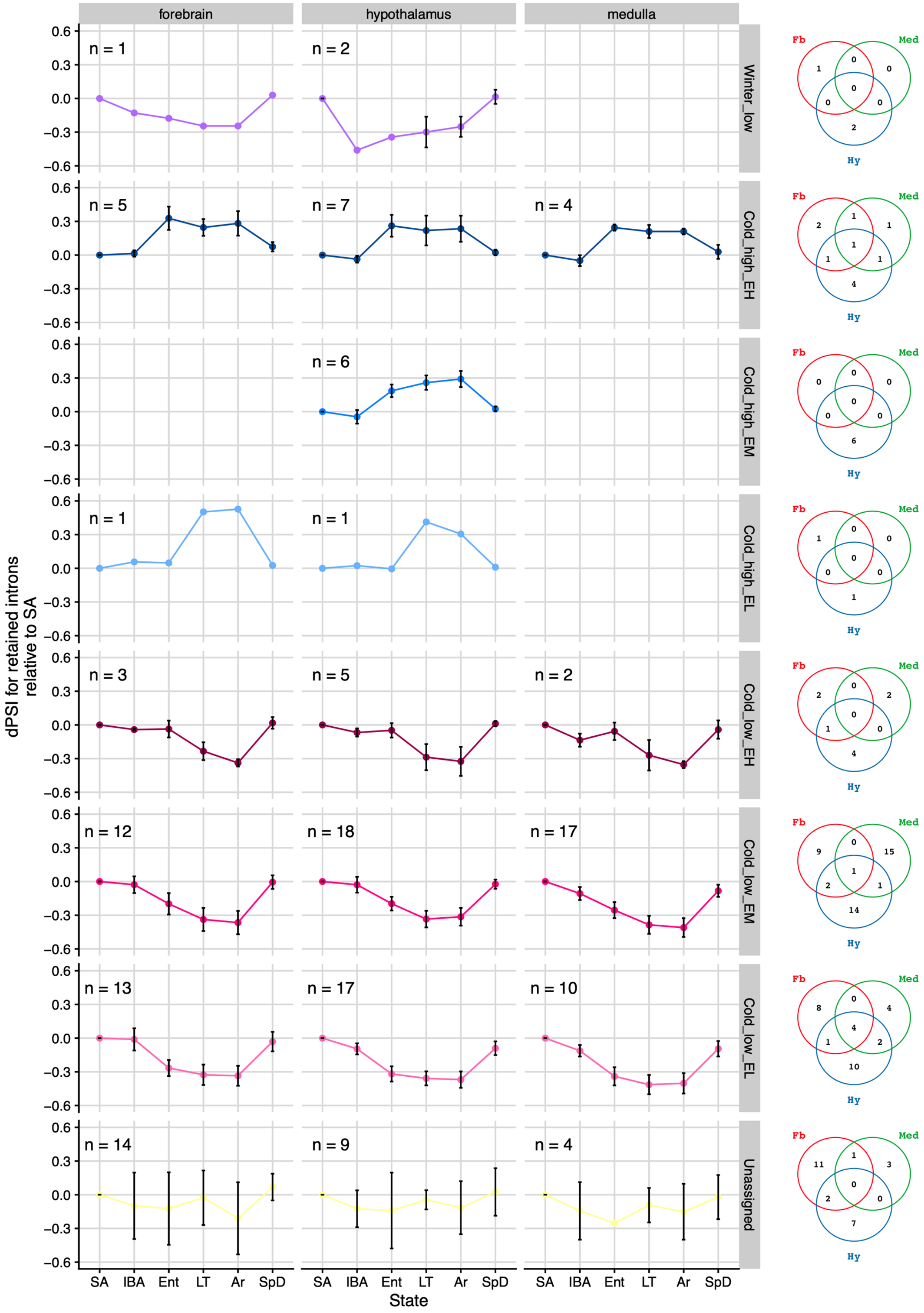

Supplementary Figure 17. Mean intron retention patterns in three brain regions. dPSI > 0 indicates increased retention of the intron relative to SA, while dPSI < 0 indicates increased excision of the intron relative to SA. Error bars indicate standard deviation. Venn diagrams to the right of each cluster quantify common and unique events among Venn diagrams quantify common and unique events among Forebrain (Fb), Medulla (Med) and Hypothalamus (Hy).

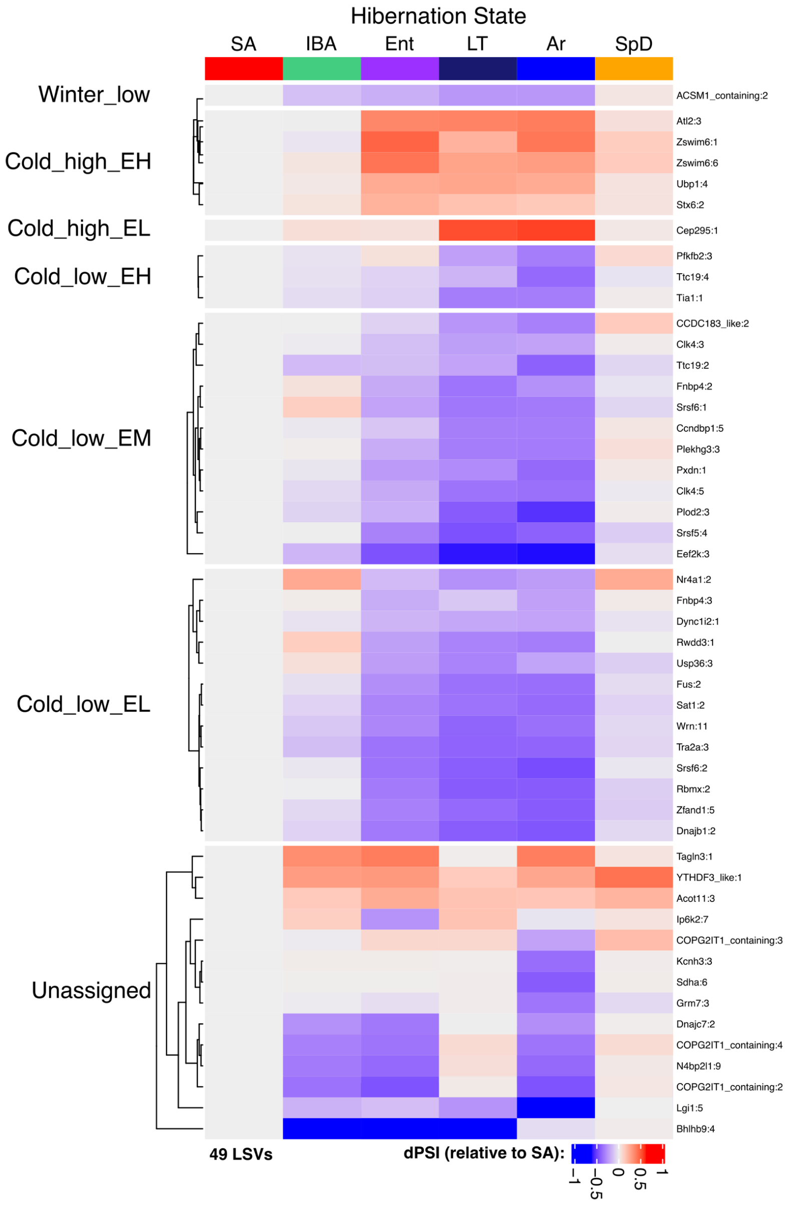

Supplementary Figure 18. Genes with retained introns in forebrain. Heatmap depicts the abundance of retained introns for each state relative to SA. dPSI > 0 means increased retention of the intron relative to SA, while dPSI < 0 means increased excision of the intron relative to SA. Gene symbols are shown to the right of the heatmap. For genes with multiple LSVs, the number is appended to the gene name, separated by a colon.

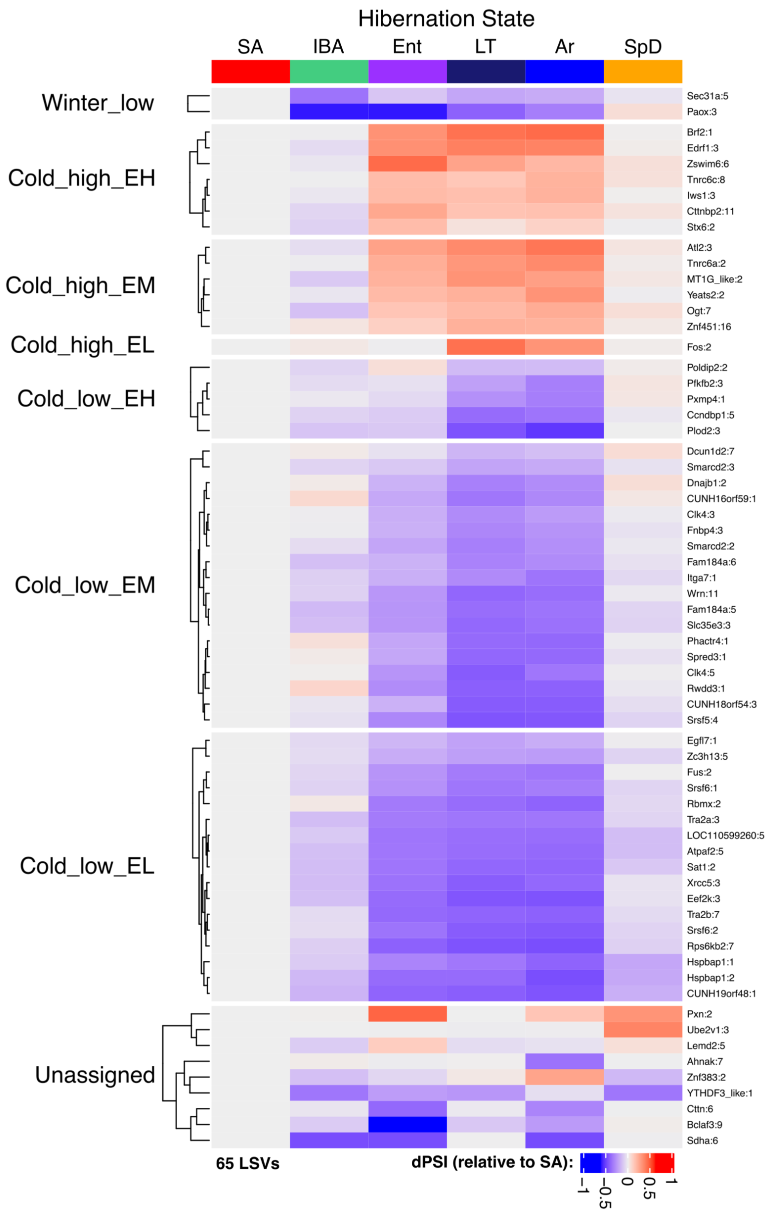

Supplementary Figure 19. Genes with retained introns in hypothalamus. Heatmap depicts the abundance of retained introns for each state relative to SA. dPSI > 0 means increased retention of the intron relative to SA, while dPSI < 0 means increased excision of the intron relative to SA. Gene symbols are shown to the right of the heatmap. For genes with multiple LSVs, the number is appended to the gene name, separated by a colon.

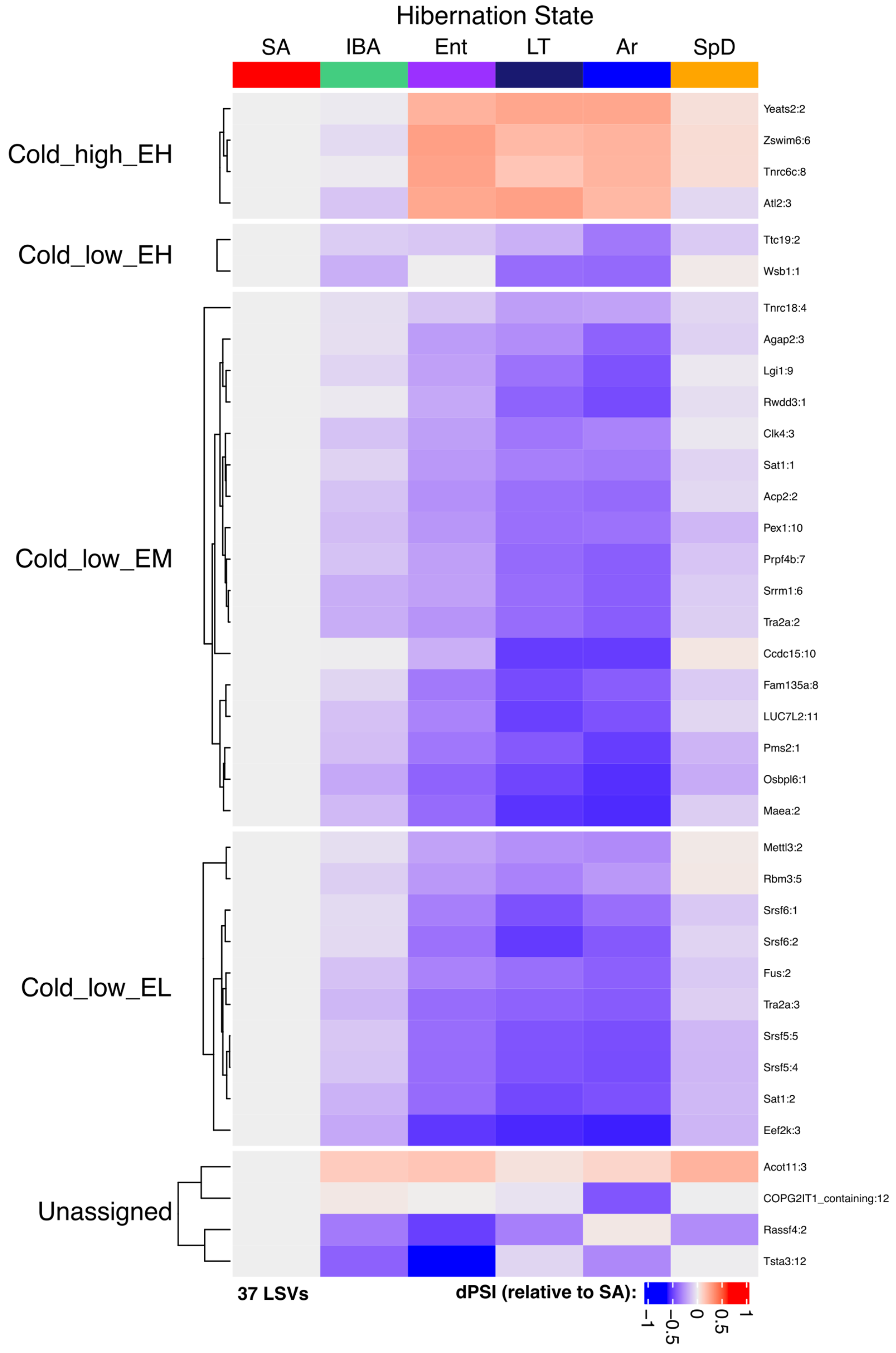

Supplementary Figure 20. Genes with retained introns in medulla. Heatmap depicts the abundance of retained introns for each state relative to SA. dPSI > 0 means increased retention of the intron relative to SA, while dPSI < 0 means increased excision of the intron relative to SA. Gene symbols are shown to the right of the heatmap. For genes with multiple LSVs, the number is appended to the gene name, separated by a colon.

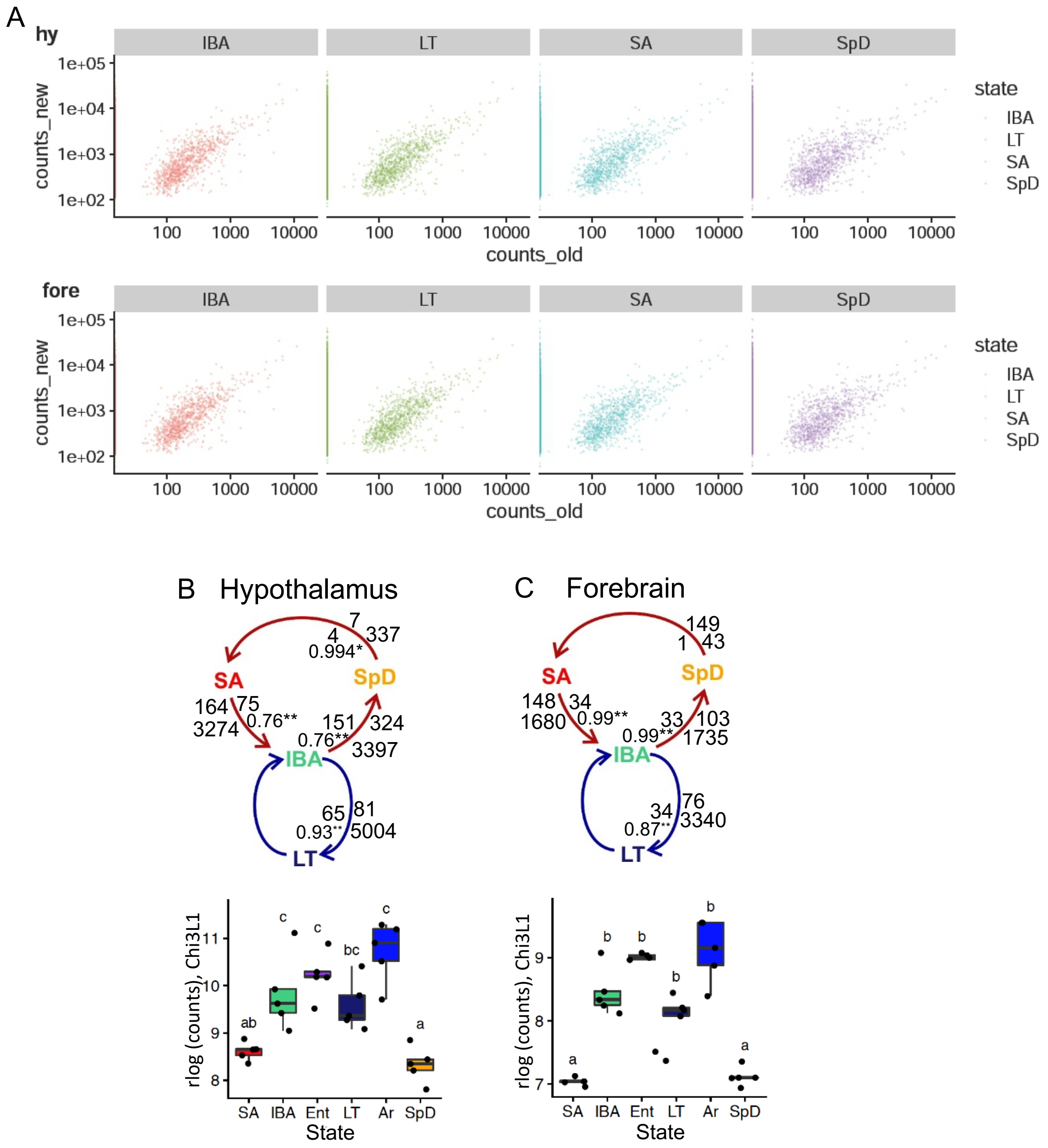

Supplementary Figure 21. Comparison of brain DE results from two studies. **A**) Gene count distributions from hypothalamus (hy) and forebrain (fore). Despite the large number of new genes and the skew to higher counts/gene, the patterns we obtained are similar to those reported in Schwartz et al., 2013. **B,C**)Top panels depict relationship of four states for which comparisons were possible from **B**) hypothalamus (both studies) and **C**) forebrain (present study) vs. cortex (in Schwartz, et al., 2013). The number (padj<0.05) of DE genes for each transition pair is shown (Ap, Oct, IBA and torpor from the previous study were taken as comparable to SpD, SA, IBA and LT from this study). Top numbers in the outside pairs are the number of DE genes from Schwartz et al., 2013, and bottom numbers are from the present data. The fold changes for DE genes common to both datasets (top of two numbers inside circles) were correlated (bottom numbers inside circles, *$p<0.05$, **$p<10^{-10}$). Bottom panels show relative abundance of Chi3L1 for our full dataset in hypothalamus (left) and forebrain (right); in both studies the pattern of expression is elevated in winter/hibernation. Letters define DE groups, dots show individual samples.

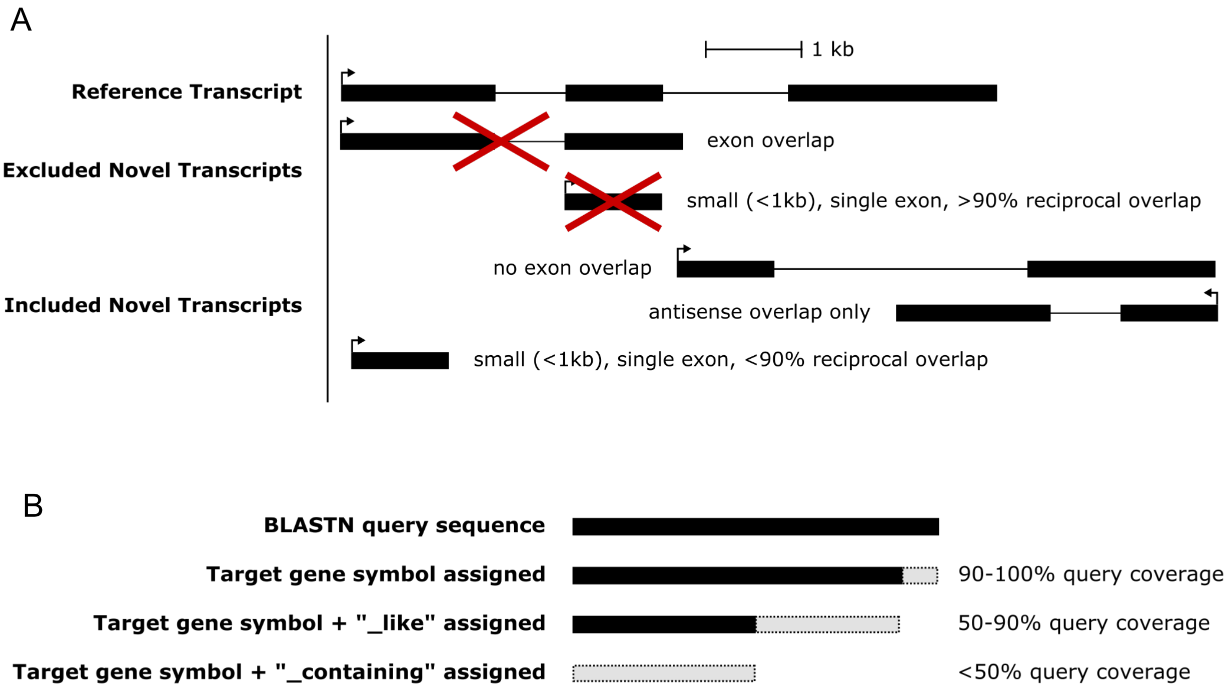

Supplementary Figure 22. Generation and refinement of the HiC_Itri_2 transcriptome annotations. **A**) Generic transcript models demonstrate how novel (or ensemble) transcripts are excluded or included in the merged transcriptome. Only transcripts with either no same-strand exon overlap or short (<1kb), single-exon transcripts with <90% reciprocal exon coverage are included. **B**) Schematic representation of the query coverage required for reannotation using BLASTN. Gene symbols for targets with e ≤ 1e-20 and >90% query coverage are assigned directly to the query transcripts, while query coverages of 50-90% and <50% result in assignment with the suffixes "_like" and "_containing", respectively.
